# Developmental Transcriptional Networks Are Required to Maintain Neuronal Subtype Identity in the Mature Nervous System

**DOI:** 10.1371/journal.pgen.1002501

**Published:** 2012-02-23

**Authors:** Kevin T. Eade, Hailey A. Fancher, Marc S. Ridyard, Douglas W. Allan

**Affiliations:** Department of Cellular and Physiological Sciences, University of British Columbia, Vancouver, Canada; University of Cambridge, United Kingdom

## Abstract

During neurogenesis, transcription factors combinatorially specify neuronal fates and then differentiate subtype identities by inducing subtype-specific gene expression profiles. But how is neuronal subtype identity maintained in mature neurons? Modeling this question in two *Drosophila* neuronal subtypes (Tv1 and Tv4), we test whether the subtype transcription factor networks that direct differentiation during development are required persistently for long-term maintenance of subtype identity. By conditional transcription factor knockdown in adult Tv neurons after normal development, we find that most transcription factors within the Tv1/Tv4 subtype transcription networks are indeed required to maintain Tv1/Tv4 subtype-specific gene expression in adults. Thus, gene expression profiles are not simply “locked-in,” but must be actively maintained by persistent developmental transcription factor networks. We also examined the cross-regulatory relationships between all transcription factors that persisted in adult Tv1/Tv4 neurons. We show that certain critical cross-regulatory relationships that had existed between these transcription factors during development were no longer present in the mature adult neuron. This points to key differences between developmental and maintenance transcriptional regulatory networks in individual neurons. Together, our results provide novel insight showing that the maintenance of subtype identity is an active process underpinned by persistently active, combinatorially-acting, developmental transcription factors. These findings have implications for understanding the maintenance of all long-lived cell types and the functional degeneration of neurons in the aging brain.

## Author Summary

For neurons to function properly, they must establish and then maintain their unique, subtype-specific gene expression profiles. These unique gene expression profiles are established during development by networks of DNA–binding proteins, termed transcription factors (TFs). However, how neurons maintain their unique gene expression profiles in the mature and aging brain is largely unknown. Recent advances in inducible genetic technologies now allow us to manipulate gene expression in adult neurons, after normal development. Applying such techniques, we examined the effect of knocking down TF expression in two adult neuronal subtypes. We show that the TF networks that establish unique gene expression profiles during development are then required to maintain them thereafter. Thus, gene expression profiles are not simply “locked-in,” but must be actively maintained by persistent developmental TF networks. However, we found that critical cross-regulatory relationships that had existed between TFs during development were not present in the adult, even between persisting TFs. This highlights important differences between developmental and maintenance transcriptional networks in individual neurons. The dependence of subtype gene expression on active mechanisms represents a potential Achilles heel for long-lived cells, as deterioration of those active mechanisms could lead to functional degeneration of neurons with advancing age.

## Introduction

Tremendous progress has been made in delineating the transcriptional mechanisms that diversify neuronal subtype identities during development. Spatiotemporally-patterned transcription factor cascades act within increasingly diversified progenitor populations to specify postmitotic neuron subtype fate. Within those postmitotic neurons, subtype-specific sets of transcription factors act combinatorially to differentiate subtype identity by initiating expression of the genes that define subtype form and function [1], [2], [3]. These so-called terminal differentiation genes include subtype-specific neuropeptides, neurotransmitter enzymes and ion channels [4]. Developmental transcriptional cascades are progressive and typically nonlinear; many transcription factors act at multiple levels, they exhibit extensive cross-regulation, and their expression undergoes considerable refinement in developing postmitotic neurons [1], [5], [6], [7]. Here, we apply the term ‘subtype transcription network’ to refer to the transcription factors that direct subtype specification and differentiation, their cross-regulatory relationships (or configuration), and the manner in which they direct the expression of subtype-specific sets of terminal differentiation genes.

After subtype-specific gene expression profiles are established by differentiation, continued neuronal function throughout life depends upon their maintenance of subtype gene expression profiles. However, we currently have only a rudimentary understanding of the mechanisms of long-term, subtype-specific gene maintenance. Two extreme models would posit that subtype identity is either actively maintained by persistent subtype transcription network activity or passively maintained by for example stabilized chromatin structure, independent of a subtype transcription network. Here, we test the active model in two *Drosophila* neuronal subtypes to address the following largely unanswered questions: Do developmental subtype transcription networks persist in adult neurons or are they dispensed with? Are they required to maintain the expression of subtype-specific sets of terminal differentiation genes? If they are required, does maintenance of terminal differentiation genes require the same complex combinatorial codes of transcription factors as for their initiation, or a simplified code involving fewer transcription factors? Finally, do persisting developmental transcription factors retain the same cross-regulatory relationships that regulated their expression during development?

We model these questions in *Drosophila* Tv1 and Tv4 neurons. There are six clusters of Tv neurons in the *Drosophila* ventral nerve cord, each comprising four distinct subtypes (Tv1–Tv4). Tv1 and Tv4 express subtype-specific terminal differentiation genes, the neuropeptides Nplp1 (Tv1) and FMRFa (Tv4) and the neuropeptide amidase PHM (peptidylglycine alpha-hydroxylating monooxygenase; in Tv1/Tv4). Using these genes as markers for subtype-specific differentiation, previous work had revealed the elaborate subtype transcription networks that direct Tv1/4 subtype specification and differentiation [6], [8], [9], [10], [11], [12], [13]. The expression of FMRFa [14], Nplp1 and PHM (herein) are stably maintained in Tv1/4 neurons throughout *Drosophila* life. Thus, our detailed understanding of their subtype-specific initiation in the embryo provides an ideal background to investigate how such terminal differentiation genes, and hence subtype identity, are maintained in the adult. We previously established that persistent retrograde BMP signaling is required to initiate and maintain FMRFa in Tv4 neurons [14]. Here, we examined the adult function of Tv1 and Tv4 subtype transcription networks in the maintenance of Nplp1, FMRFa and PHM. We further examined whether the cross-regulatory interactions observed between the Tv1/Tv4 network transcription factors during development were maintained in adults. We found that each subtype transcription network is largely retained in adult Tv neurons and is required to actively maintain subtype-specific gene expression. Thus, the combinatorial transcription codes for subtype-specific gene expression are not ‘simplified’ or dispensed with for maintenance. Further, we find that certain critical developmental cross-regulatory interactions between transcription factors are no longer utilized in adults for transcription factor maintenance. Thus, we observe a post-developmental switch to a distinct maintenance configuration between individual transcription factors. Collectively, these data provide novel insight relevant to understanding how long-lived cell types maintain their subtype identity.

## Results

**Figure 1:**
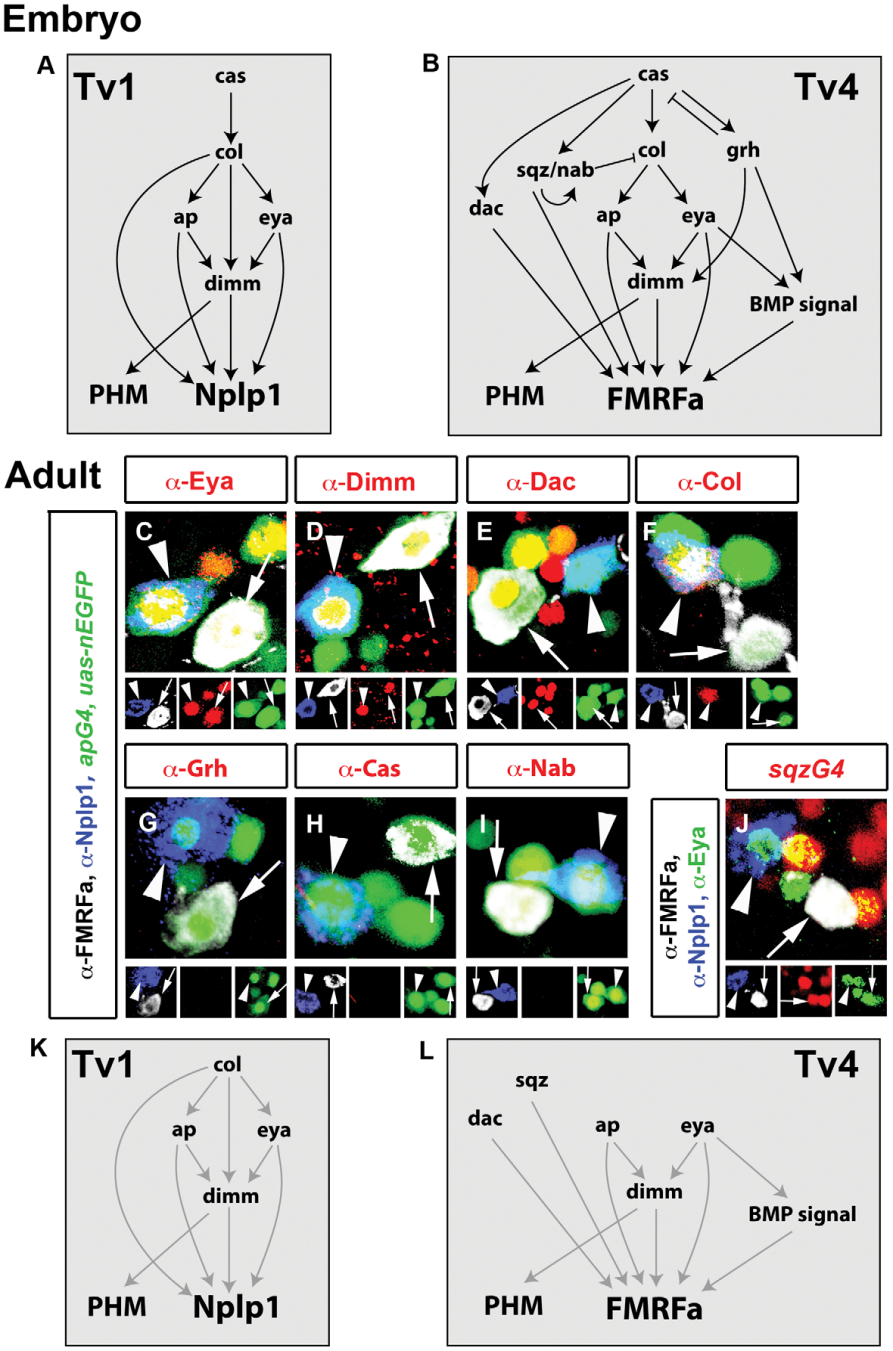
Adult Tv1 and Tv4 neurons maintain Nplp1 and FMRFa and a subset of embryonic transcription factors. (A–B) Outline of transcription factor network configuration during specification and differentiation of Tv1 (A) and Tv4 (B) neurons at embryonic stages. Tv1 expresses Nplp1 (blue). Tv4 expresses FMRFa (red). Tv1 and Tv4 neurons both express PHM. Arrows represent known regulatory relationships. (C–J) Representative images from Tv clusters in adults (green) in thoracic hemisegments 1 and 3. Tv4 neurons (arrows) express FMRFa (white). Tv1 neurons (arrowheads) express Nplp1 (blue). Tv1 and Tv4 retain expression of transcription factors *ap* (C–I, green), *eya* (C red, J green) and *dimm* (D, red). Tv1 neurons also retains *col* (F, red). Tv4 neurons also retain *dac* (E, red) and *sqz* (J, red). Tv1 and Tv4 do not express transcription factors *grh* (G, red), *cas* (H red) or *nab* (I, red) in adults. Genotype: (C–I) *FMRFa-LacZ,ap^Gal4^/+; UAS-nEGFP/+*. (J) *UAS-nEGFP/+*; sqz*^Gal4^/+*. (K,L) Outline of transcription factors present in fully differentiated adult Tv1 (K) and Tv4 (L) neurons. Grey arrows indicate known developmental interactions between transcription factors in embryonic Tv cluster neurons that, herein, we test in the adult.

Genetic analysis has defined cascades of transcription factors that specify Tv1 and Tv4 neuron fates and then differentiates their subtype-specific terminal differentiation gene expression profiles (Figure 1A, 1B) [6], [8], [9], [10], [11], [12]. Tv1–4 neurons are born sequentially from the NB5-6T neuroblast lineage within an expression window of the ‘temporal’ transcription factors *castor* (*cas*) and *grainy head* (*grh*) [6], [15]. These specify Tv subtype generation together with *collier* (*col*), *squeeze* (*sqz*) and *nab* [6], [9], [12], [16]. Within postmitotic Tv1 neurons, *ap*, *eya*, *dimmed (dimm)* and *col* then differentiate Tv1 identity in part by initiating Nplp1 expression [9]. In Tv4 neurons *ap*, *eya*, *dimm*, *dachshund (dac)*, *sqz* and *grh* act combinatorially with target-derived BMP signaling to differentiate Tv4 identity, in part by initiating FMRFa expression [6], [10], [11], [12], [17], [18], [19]. Also, in both Tv1 and Tv4, *dimm* acts independently of other regulators to induce expression of the neuropeptide amidase, PHM [10], [20]. We refer to these two transcription factor cascades as the Tv1 and Tv4 ‘subtype transcription networks’ (outlined in Figure 1A, 1B). Genetic analysis has placed these transcription factors into two partially overlapping categories; those that are necessary for directing the specification, or generation, of Tv subtypes around the time of postmitotic neuron birth, and those that thereafter direct differentiation of Tv subtypes by initiating subtype-specific terminal differentiation gene expression. Tv1 neurons are not specified (or generated) in *cas* or *col* mutants, whereas *eya*, ap, *col* and *dimm* all act combinatorially thereafter to initiate Nplp1 expression [6], [9]. Tv4 neurons are not specified in *cas*, *col* or *grh* mutants. In *sqz* and *nab* mutants, Tv4 neurons are not specified in a segment-specific manner [6], [12]. Thereafter, *eya*, *ap*, *sqz*, *dimm*, *dac*, *grh* and BMP signaling all appear to combinatorially initiate FMRFa expression.

### Subtype transcription network retention in adult Tv1 and Tv4 neurons

We previously reported that adult Tv4 neurons maintain FMRFa, *ap* and *eya* expression, as well as retrograde BMP-signaling. We also demonstrated that FMRFa maintenance in adult Tv4 neurons requires persistent retrograde BMP-signaling [14]. Here, we determined the adult expression of the other transcriptional regulators implicated in Tv1 and Tv4 development (Figure 1C–1J). We found that adult Tv4 neurons retained *ap*, *eya*, *dimm*, *dac* and *sqz* (Figure 1C–1E, 1J), but no longer expressed *grh*, *cas* or *nab* (Figure 1G–1I). Additionally, adult Tv1 neurons retained expression of Nplp1, as well as *ap*, *eya*, *dimm* and *col* (Figure 1C, 1D, 1F). Previously, *cas* expression was shown to be lost in all Tv neurons prior to neuropeptide initiation [9], and here we find that it does not become re-expressed in adult Tv1 or Tv4 neurons (Figure 1H). These data are summarized (Figure 1K, 1L). Further analysis found that the adult complement of transcription factors was established by the start of the L1 larva stage (Figure S1), shortly after Tv1/4 terminal differentiation.

Which transcription factors persist in adult neurons is intriguing. All those previously implicated in the postmitotic differentiation of Tv1 and/or Tv4 neurons persist. In contrast, transcription factors that act within the neuroblast and newborn postmitotic neuron to specify the fate of Tv1 (*cas*) or Tv4 (*cas, col, grh, nab*) neurons are not retained in the adult. The exceptions to this are *col* and *sqz*. Both are implicated in Tv subtype specification, but it is notable that both transcription factors have also been implicated, by loss and gain of function genetics, as part of the combinatorial transcription factor codes that initiate Nplp1 or FMRFa expression [6], [9], [12]. Thus, we find that only regulators implicated in postmitotic subtype differentiation are maintained in adult neurons.

### Conditional *dsRNAi* knockdown in adult Tv neurons

**Figure 2:**
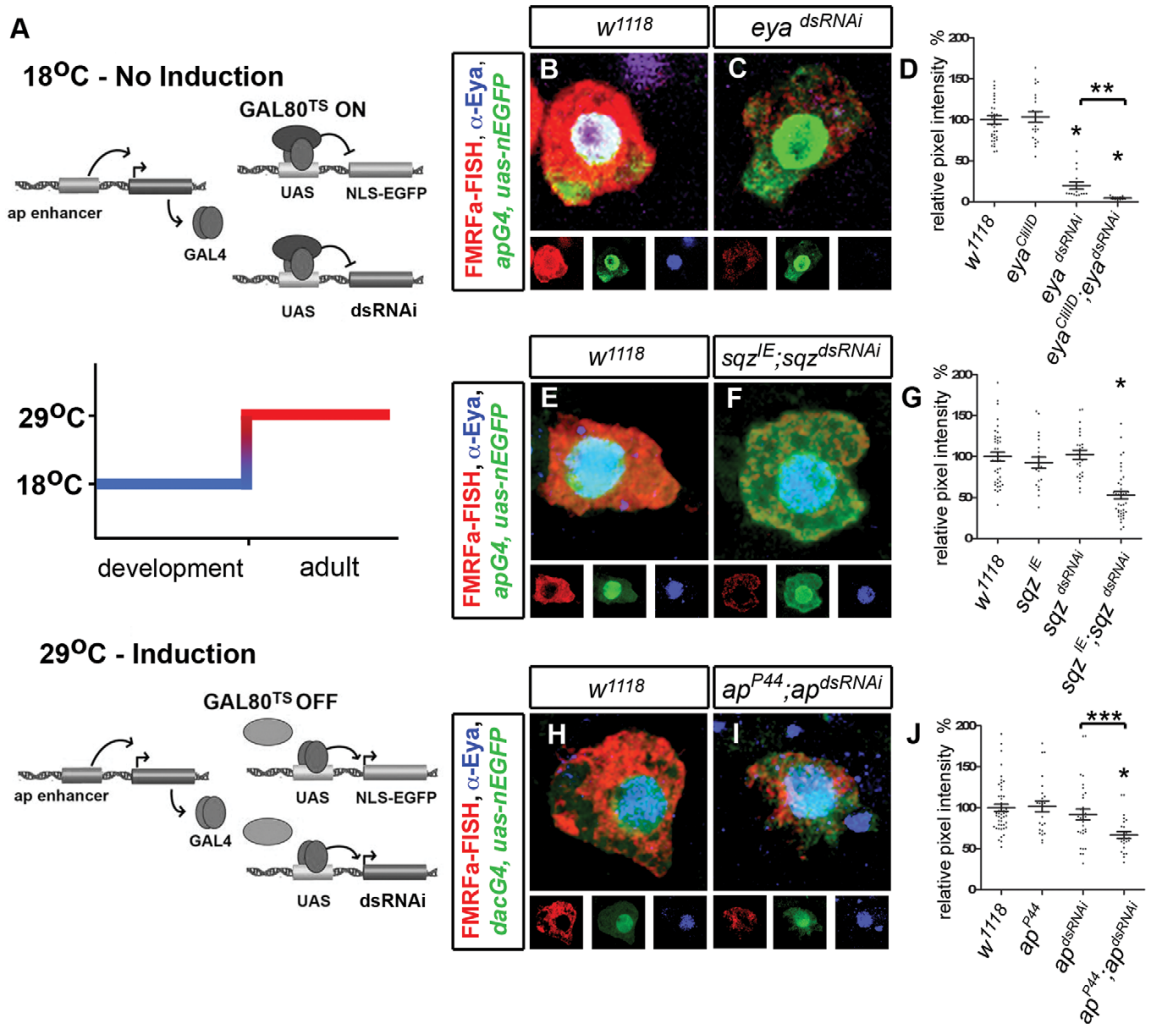
Transcription factors *ap*, *eya*, *sqz* are required for persistent FMRFa expression in the adult Tv4 neuron. (A) Cartoon illustrating adult induction of dsRNAi constructs in adult Tv neurons using the TARGET system. (B,C, E,F, H,I) Images of adult Tv4 neurons expressing FMRFa transcript (red), *ap^Gal4^,UAS-nlsEGFP* (green) and Eya (blue) after A15 (B–G) or A20 (H–J) of *dsRNAi* expression at 29°C. (B,C) Eya immunoreactivity is eliminated and FMRFa is downregulated in *eya^dsRNAi^* (C) compared to *w^1118^* control (B). (E,F) FMRFa is downregulated in *sqz^IE^*,*sqz^dsRNAi^* (F) compared to *w^1118^* control (E). (H,I) FMRFa is downregulated in *ap^P44^;ap^dsRNAi^* (I) compared to *w^1118^* control (H). (D,G,J) Quantification of FMRFa transcript in individual adult Tv4 neurons at A15 (D,G) or A20 (J) at 29°C. (D) * p<0.0001 eya*^dsRNAi^* (n = 15) and *eya^dsRNAi^; eya^CliIID^* (n = 13) compared to *w^1118^* (n = 35) and *eya^CliIID^* controls. ** p<0.0001 compared to eya*^dsRNAi^* alone. (G) * p<0.0001 *sqz^IE^; sqz^dsRNAi^* (n = 39) compared to *w^1118^* (n = 40), *sqz^IE^* (n = 21), and *sqz^dsRNAi^* (n = 25) controls. (J) * p<0.0001 *ap^P44^; ap^dsRNAi^* (n = 25) compared to *w^1118^* (n = 47) and *ap^P44^* (n = 25) controls. *** p<0.005 compared to ap*^dsRNAi^* (n = 32) alone. *Genotypes*: *w^1118^* (*UAS-dicer2/+; ap^Gal4^/+; tub-Gal80^TS^, UAS-nEGFP*) (A–C) *eya^CliIID^* (*UAS-dicer2/+; ap^Gal4^/eya^CliIID^; tub-Gal80^TS^, UAS-nEGFP/+*); *eya^dsRNAi^* (*UAS-dicer2/UAS-eya^dsRNAi^; ap^Gal4^/+; tub-Gal80^TS^, UAS-nEGFP/+*); *eya^dsRNAi^; eya^CliIID^* (*UAS-dicer2/UAS-eya^dsRNAi^; ap^Gal4^/eya^CliIID^; tub-Gal80^TS^, UAS-nEGFP/+*). (E–G) *sqz^IE^* (*UAS-dicer2/+; ap^Gal4^/sqz^IE^; tub-Gal80^TS^, UAS-nEGFP/+*); *sqz^dsRNAi^* (*UAS-dicer2/+; ap^Gal4^/+; tub-Gal80^TS^, UAS-nEGFP/UAS-sqz^dsRNAi^*); *sqz^IE^; sqz^dsRNAi^* (*UAS-dicer2/+; ap^Gal4^/sqz^IE^; tub-Gal80^TS^, UAS-nEGFP/UAS-sqz^dsRNAi^*). (H–I) *ap^P44^* (*UAS-dicer2/+; ap^Gal4^/ap^P44^; tub-Gal80^TS^, UAS-nEGFP/+*); *ap^dsRNAi^* (*UAS-dicer2/+; ap^Gal4^/+; tub-Gal80^TS^, UAS-nEGFP/UAS-ap^dsRNAi^*); *ap^P44^; ap^dsRNAi^* (*UAS-dicer2/+; ap^Gal4^/ap^P44^; tub-Gal80^TS^, UAS-nEGFP/UAS-ap^dsRNAi^*).

To test the function of each transcription factor in maintaining terminal differentiation gene expression in Tv1 and Tv4 neuronal subtypes, we used *ap^GAL4^* (except where noted) to express *UAS-dsRNAi* transgenes (abbreviated to *dsRNAi*) targeted to each transcription factor. We also overexpressed *UAS-Dicer2* in all experiments to enhance *dsRNAi* efficacy [21]. To selectively induce *dsRNAi* in adults, we utilized the TARGET system wherein a temperature-sensitive GAL4-repressor GAL80 (GAL80^TS^) controls the activity of GAL4 [22]. Flies were raised at 18°C to allow functional GAL80^TS^ to repress GAL4 activity throughout development. Then at adult day 1 (A1), flies were switched to 29°C and kept at that temperature for the remainder of each experiment. At this temperature, GAL80^TS^ becomes dysfunctional and thus GAL4 is allowed to induce *dsRNAi* expression (Figure 2A) [14].

### *eya*, *ap*, and *sqz* are required for FMRFa maintenance in adults

We induced *eya^dsRNAi^* expression at adult day 1 (A1) and quantified FMRFa transcript levels by fluorescent *in situ* hybridization, relative to the mean of controls. In adults, *eya^dsRNAi^* dramatically reduced FMRFa transcript to 19.7±4.4% of control (p<0.0001) by adult day A15 (Figure 2B–2D). We observed a similar downregulation in immunoreactivity to the mature amidated FMRFa peptide (Table S1). To confirm *dsRNAi* specificity, we tested for enhancement of the FMRFa phenotype when expressing *eya^dsRNAi^* in an *eya* heterozygous background. Indeed, we found that this further reduced FMRFa transcript to 4.6±0.5% of control by A15 (p<0.0001 to *eya^dsRNAi^* alone or *eya* heterozygosity alone) (Figure 2D). Eya immunoreactivity was eliminated in all cases (Table S4). Previous studies demonstrated that FMRFa is severely downregulated in *eya* mutants by late embryogenesis [11]. Our data now show that FMRFa maintains this critical dependence on *eya* in adults.

We next tested the role of *apterous* (*ap*). As *ap^GAL4^* is a strong hypomorphic *ap* allele [23], we used *dac^GAL4^* to express *ap^dsRNAi^* in wildtype and heterozygous *ap* backgrounds. We observed a significant downregulation of FMRFa transcript when expressing *ap^dsRNAi^* in *ap* heterozygotes, falling to 66.8±4.2% of control (p<0.0001 from control or *ap* heterozygote alone, and p<0.005 from *ap^dsRNAi^* alone). No loss of FMRFa was observed in either *ap* heterozygotes or *ap^dsRNAi^* alone (Figure 2H–2J). Similar results were obtained for downregulation of the mature FMRFa amidated peptide (Table S1). We also examined FMRFa expression using the strong *ap^GAL4^* driver to overexpress *ap^dsRNAi^*, and observed a significant reduction of mature amidated FMRFa peptide to 40.0±2.2% of control by A20 (*w^1118^* control n = 40, *ap^dsRNAi^* n = 38; p<0.0001). Finally, we also confirmed that an *ap^dsRNAi^* targeting different *ap* sequences also significantly downregulated FMRFa (Table S2). The downregulation of FMRFa that we observed in adults is comparable to that reported for embryonic *ap* null mutants [12], [17]. Thus, we conclude that *ap* maintains a persistent role in FMRFa regulation. We were unable to determine the extent of Ap knockdown by either *ap^dsRNAi^* transgene due to a lack of suitable Ap-specific antibodies. Therefore, we tested *ap^dsRNAi^* efficacy by examining another *ap* phenotype. In *ap* mutants, the wings fail to form [24]. We found that expression of *ap^dsRNAi^* in the developing wing, using *ap^GAL4^*, could precisely phenocopy this *ap* phenotype (Figure S2). Thus, we conclude that *ap^dsRNAi^* is specific and highly effective. However, as we could not directly quantify Ap downregulation in Tv neurons, we cannot formally discount the possibility that FMRFa would be further downregulated if Ap were entirely eliminated.

To test the role of *sqz* in adult Tv4 neurons, we expressed *sqz^dsRNAi^* at A1 and observed a partial downregulation of FMRFa expression in *sqz* heterozygotes to 53.0±4.8% of control (p<0.0001 from control, *sqz* heterozygote or *sqz^dsRNAi^* alone) (Figure 2E–2G). Similar results were obtained for immunoreactivity to the mature amidated FMRFa peptide (Table S1). Previous reports established that FMRFa is partially downregulated in embryonic *sqz* mutants [12]. Thus, our data indicate that *sqz* maintains its partial requirement for FMRFa expression. Due to ubiquitous but weak Sqz expression in the thoracic nerve cord, we were not able to adequately quantitate Sqz downregulation in Tv neurons. Thus, we do not discount the possibility that Sqz may not have been entirely eliminated, and therefore we may be underestimating its effect on FMRFa expression. Taken together, our data demonstrate that *eya*, *ap* and *sqz* are required to maintain wildtype FMRFa levels in the adult.

### *dimm* maintains FMRFa peptide processing

**Figure 3:**
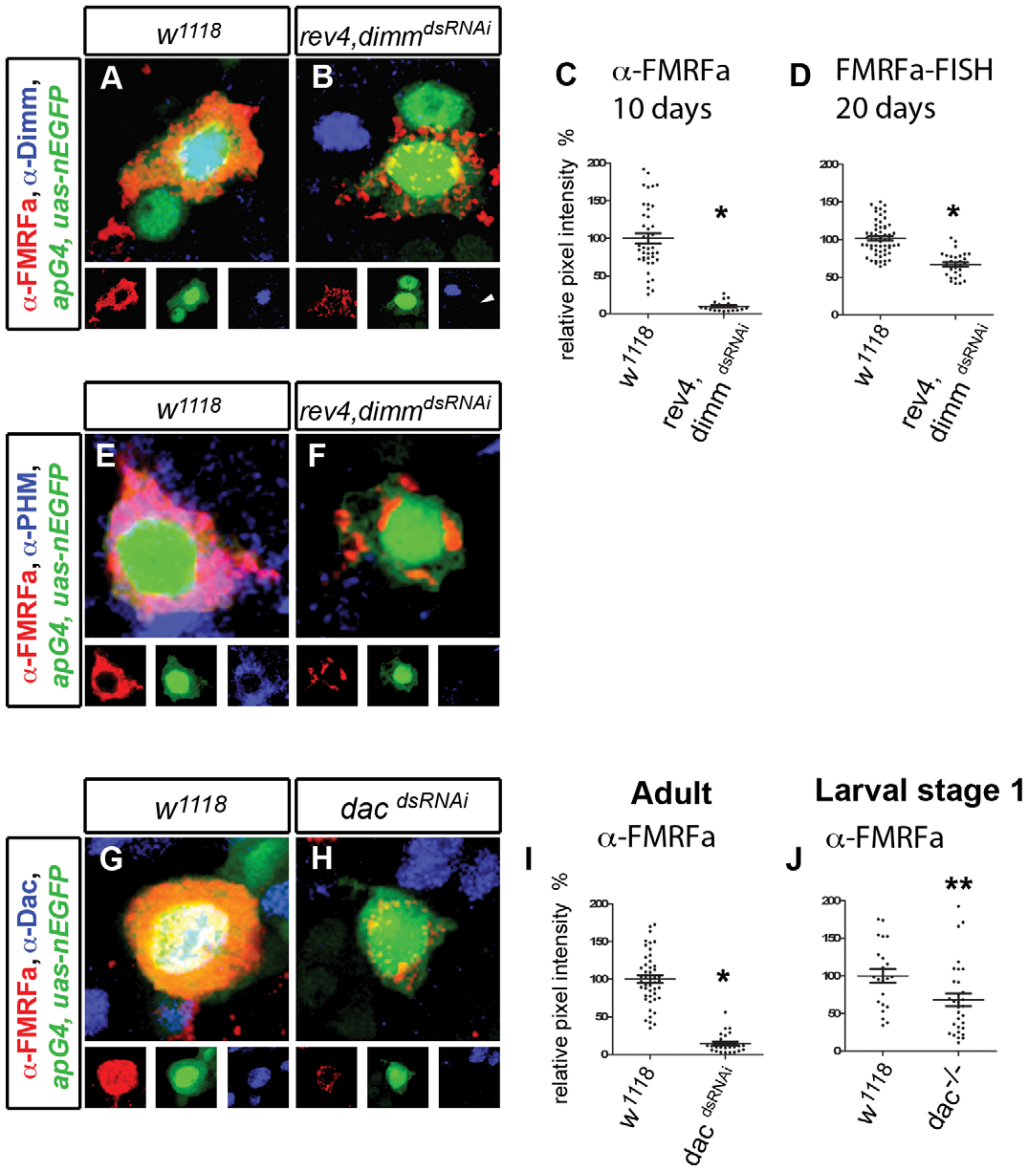
*dimm* maintains peptidergic phenotype and *dac* has an enhanced maintenance function. (A,B) Representative images of adult Tv4 neurons expressing FMRFa peptide (red), *ap^Gal4^,UAS-nlsEGFP* (green) and Dimm (blue) at A10 at 29°C. FMRFa is downregulated and Dimm is lost in *rev4,dimm^dsRNAi^* (B) compared to *w^1118^* control (A). (C,D) Quantification of FMRFa peptide at A10 (C) and FMRFa transcript at A20 (D) in individual adult Tv4 neurons at 29°C. (C)* p<0.0001 *rev4,dimm^dsRNAi^* (n = 19) compared to *w^1118^* control (n = 42). (D)* p<0.0001 *rev4,dimm^dsRNAi^* (n = 30) compared to *w^1118^* control (n = 58). (E,F) Representative images of Tv4 neurons expressing mature FMRFa peptide (red), *ap^Gal4^,UAS-nlsEGFP* (green) and PHM (blue) in adult Tv4 neurons at A10 at 29°C. PHM is lost in *rev4,dimm^dsRNAi^* (n = 26) (F) compared to *w^1118^* control (n = 30) (E). (G,H) Images of adult Tv4 neurons expressing FMRFa peptide (red), *ap^Gal4^,UAS-nEGFP* (green) and Dac (blue) at A10 at 29°C. FMRFa is downregulated and Dac immunoreactivity is lost in *dac^dsRNAi^* (H) compared to *w^1118^* control (G). (I,J) Quantification of FMRFa peptide in individual adult Tv4 neurons at A10 at 29°C (I), and in L1 larval Tv4 neurons in *dac* null mutants. (J) * p<0.0001 *dac^dsRNAi^* (n = 26) compared to *w^1118^* control (n = 47). (J) ** P = 0.02 *dac^−/−^* (n = 31) compared to *w^1118^* control (n = 22). *Genotypes*: *w^1118^* (*UAS-dicer2/+; ap^Gal4^/+; tub-Gal80^TS^, UAS-nEGFP/+*); *rev4,dimm^dsRNAi^* (*UAS-dicer2/+; ap^Gal4^/rev4, UAS-dimm^dsRNAi^; tub-Gal80^TS^, UAS-nEGFP/+*); *dac^dsRNAi^* (*UAS-dicer2/+; ap^Gal4^/UAS-dac^dsRNAi^; tub-Gal80^TS^, UAS-nEGFP/+*); *dac^−/−^* (*dac^3^/dac^Df(3L)EXEL 7066^*).

We induced *dimm^dsRNAi^* at A1 and found that immunoreactivity to the mature amidated FMRFa peptide was rapidly and profoundly reduced by *dimm^dsRNAi^* to 24.0±3.2% of control by A10 (p<0.0001), and this was enhanced to 9.8±1.6% of control in *dimm* heterozygotes (p<0.0001 to control and *dimm* heterozygotes, p<0.001 to *dimm^dsRNAi^* alone) (Figure 3A–3C, Figure S3). Immunoreactivity to Dimm demonstrated that it had been eliminated (Table S4). In contrast, FMRFa transcript in adults was downregulated in *dimm* heterozygotes to 67.1±2.9% of control at A20 (p<0.0001) (Figure 3D). Similar effects were observed using a *dimm^dsRNAi^* that targets different *dimm* sequences (Table S2). It is notable that downregulation of the transcript was only observed after 20 days of *dimm^dsRNAi^* induction but the peptide was profoundly reduced after only 10 days of induction. In late Stage 17 embryonic *dimm* mutants, immunoreactivity to the mature amidated FMRFa peptide was profoundly reduced, but the extent to which FMRFa transcript was affected had not been quantified [10], [18]. Here, we find that FMRFa transcript was only modestly downregulated in late Stage 17 embryonic *dimm* mutants to 71.6±3.9% of controls (wild type control n = 54, *dimm* mutant n = 34 (p<0.0001)). Thus, we conclude that *dimm* retains its role in the initiation and maintenance of both FMRFa transcript and mature peptide. Why is the mature peptide more responsive to *dimm^dsRNAi^* than is the transcript? We postulated that this was due to *dimm*'s regulation of proprotein convertases and peptide amidases in secretory neurons, both of which are required to process the FMRFa prepropeptide into amidated neuropeptides [10], [20], [25]. We tested this in adults by examining expression of peptidylglycine α-hydroxylating monooxygenase after *dimm^dsRNAi^* induction (PHM). Confirming our hypothesis, *dimm^dsRNAi^* entirely eliminated PHM immunoreactivity in Tv4 neurons (Figure 3E, 3F). Thus, the maintenance of neuropeptide-processing enzyme expression and biosynthesis of the amidated FMRFa peptide is highly dependent upon persistent *dimm* function in adult Tv neurons.

### *dac* is necessary for FMRFa maintenance

Previous studies found that FMRFa was only modestly downregulated in *dac* mutants during development [11]. In confirmation, we found here that in L1 larvae, FMRFa immunofluorescence per Tv4 neuron was 68.3±8.5% of control (Figure 3J; P<0.02). We tested *dac* function in adults and found that *dac^dsRNAi^* dramatically downregulated FMRFa immunoreactivity in adults to 14.8±2.5% of controls, as early as A10 (p<0.0001) (Figure 3G–3I). Correspondingly, FMRFa transcript was reduced to 24.9±4.2% of controls (p<0.0001 to controls), and this was enhanced in *dac* heterozygotes to 6.5±0.8% (p<0.001 to *dac^dsRNAi^* alone) (Figure S3). Notably, by A15, FMRFa peptide and transcript were entirely eliminated (not shown). In all cases, we found that Dac immunoreactivity was eliminated (Table S4). Moreover, similar effects were observed using a *dac^dsRNAi^* that targets different *dac* sequences (Figure S2). Thus, *dac* appears to be unique amongst the Tv4 subtype transcription network factors in that it assumes an increasingly essential role in maintenance compared to developmental initiation.

### Post-developmental changes to Tv4 subtype transcription network configuration

The Tv4 subtype transcription network acts through hierarchical and feedforward transcription factor activity, which we refer to here as the network's configuration (summarized in Figure 1A, 1B) [6], [10]. Initiation of *grh*, *dac*, *sqz* and *col* requires transient *cas* activity [6]. Expression of *ap* and *eya* requires transient *col* expression [9]. The induction of *dimm* then requires *eya*, *ap* and *grh* [6], [9], [10]. BMP signaling is dependent upon *eya* [11]. Finally, *ap*, *eya*, *dimm*, *dac*, *sqz*, *grh* and BMP signaling are all required for FMRFa initiation [6], [11], [12], [17], [18]. This cascade represents a progressive and dynamic set of interactions during Tv4 neuron specification and differentiation. However, for long-term maintenance of subtype gene expression, the subtype transcription network presumably resolves into a stable configuration. As *grh*, *cas* and *col* are lost by early L1 (Figure S1), network configuration must change as the remaining transcription factors become independent of those that initiated their expression. However, we wished to ask whether the developmental cross-regulatory interactions between the persisting transcription factors are retained in the adult to help stabilize the network post-developmentally. Thus, we examined the configuration (cross-regulatory interactions) of all Tv4 subtype transcription network factors.

**Figure 4:**
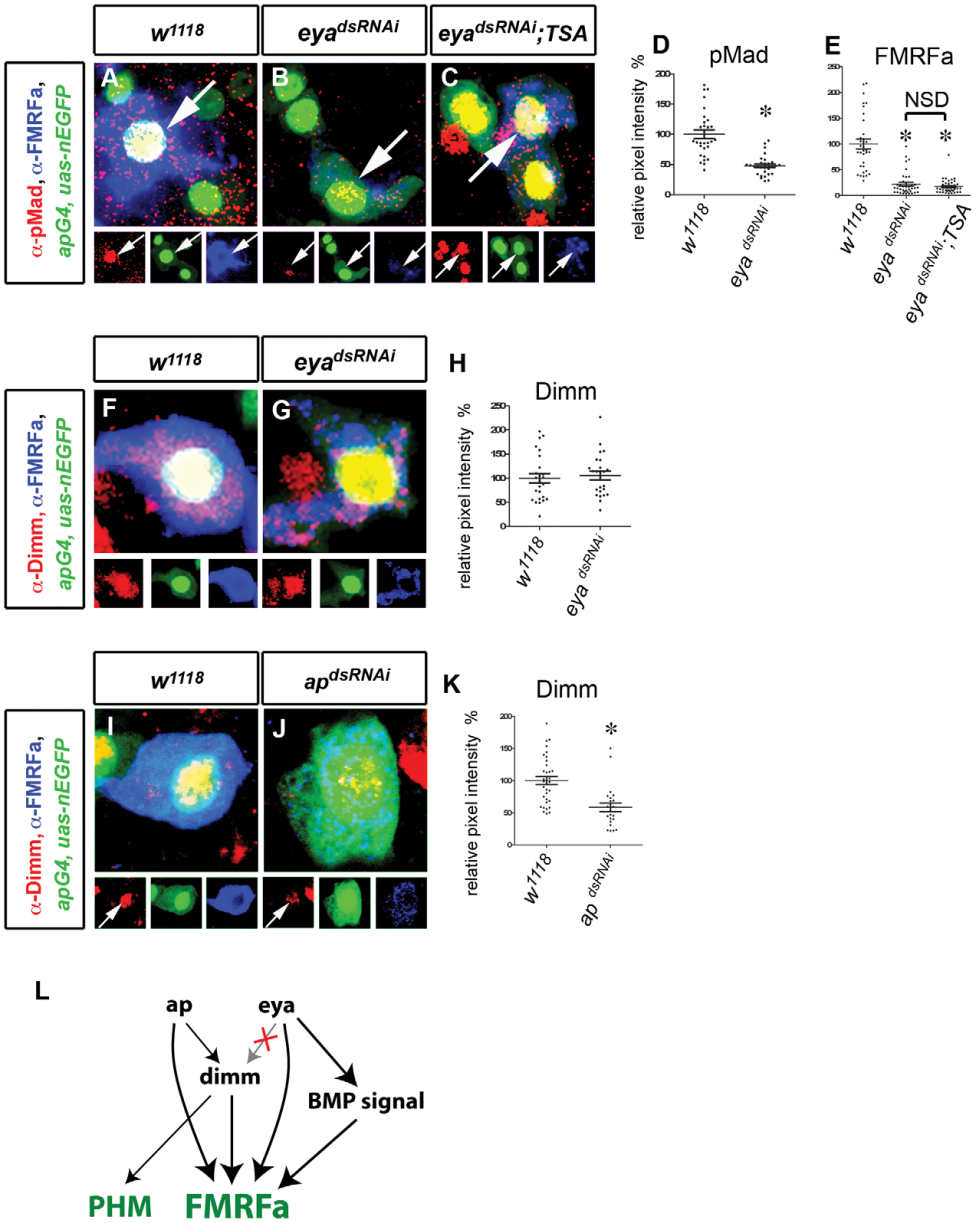
Changes in Tv4 network configuration for maintenance. (A–E) *eya* regulates FMRFa independently of BMP signaling. (A,B) Nuclear pMad accumulation (red) in Tv4 (arrowhead) was downregulated in *eya^dsRNAi^* flies (B) compared to *w^1118^* control (A). (C) Expression of *eya^dsRNAi^* in the presence of constitutively-activated Thickveins and Saxophone BMP type I receptors (*TSA*) activated pMad accumulation (red) in all Tv neurons (including Tv4; arrow) but failed to rescue FMRFa (blue) compared to *w^1118^* control. (D) Quantification of pMad immunoreactivity in Tv4 nucleus in *w^1118^* and *eya^dsRNAi^* flies. * p<0.0001 eya*^dsRNAi^* (n = 30) compared to *w^1118^* control (n = 30). (E) Quantification of FMRFa immunoreactivity in Tv4 in *w^1118^*, *eya^dsRNAi^* and *eya^dsRNAi^*; *TSA*. * p<0.0001 eya*^dsRNAi^* (n = 38) and *eya^dsRNAi^*; *TSA* (n = 42) compared to *w^1118^* control (n = 34). NSD, no significant difference between eya*^dsRNAi^* and *eya^dsRNAi^*; *TSA*. (F–H) *eya* does not regulate *dimm* in adult Tv4. *dimm* (red) in Tv neurons (blue) is maintained in *eya^dsRNAi^* (G) compared to *w^1118^* control (F). (H) Quantification of Dimm immunoreactivity in Tv4. There is no significant difference between *eya^dsRNAi^* (n = 24) and *w^1118^* (n = 25) controls (p = 0.67). (I–K) *ap* regulates *dimm* in adult Tv4. *dimm* (red) in Tv neurons (blue) is downregulated in *ap^dsRNAi^* (J) compared to *w^1118^* control (I). (K) Quantification of Dimm immunoreactivity in Tv4. * p<0.0001 *ap^dsRNAi^* (n = 24) compared to *w^1118^* (n = 35). (L) Model depicting regulatory configuration of *ap*, *eya*, *dimm* and BMP signaling from embryonic to adult Tv4. The dependence of *dimm* on *eya* expression is not maintained (X). *Genotypes*: *w^1118^* (A,D, F,H, I,J) (*UAS-dicer2/+; ap^Gal4^/+; tub-Gal80^TS^, UAS-nEGFP/+*); *eya^dsRNAi^* flies (B,D,E, G,H) (*UAS-dicer2/UAS-eya^dsRNAi^; ap^Gal4^/+; tub-Gal80^TS^, UAS-nEGFP/+*); *eya^dsRNAi^; TSA* flies (C,E) (*UAS-dicer2/UAS-eya^dsRNAi^; ap^Gal4^/UAS-tkv^A^, UAS-sax^A^; tub-Gal80^TS^, UAS-nEGFP/+*). *ap^dsRNAi^* flies (J),K (*UAS-dicer2/+; ap^Gal4^; tub-Gal80^TS^, UAS-nEGFP/UAS-ap^dsRNAi^*).

BMP signaling in embryonic Tv4 neurons is dramatically reduced in *eya* mutants [11]. We expressed *eya^dsRNAi^* in adults until A15 and found that nuclear pMad, an indicator of BMP activity [12], was significantly downregulated to 47.9%±2.9 of control (Figure 4A, 4B, 4D). As BMP signaling is required for FMRFa expression in embryos and adults, we asked whether *eya*-dependence of FMRFa in adults is due to reduced BMP signaling. To do this, we simultaneously expressed *eya^dsRNAi^* and restored BMP signaling, using constitutively-activated type I BMP-receptors, *thickveins* and *saxophone*. Even though nuclear pMad was robustly activated in all Tv neurons, *eya^dsRNAi^*-induced FMRFa downregulation was not rescued (Figure 4C, 4E). Thus, in adults, *eya* independently maintains both BMP signaling and FMRFa expression.

In the embryo, initiation of *dimm* expression in Tv4 is absolutely dependent upon *eya* and *grh* [6], [9] and partially dependent upon *ap* [10]. As *grh* is not expressed in adult Tv4 neurons, *dimm* maintenance must become independent of *grh*. However, as *eya* and *ap* are retained, we tested their role in *dimm* maintenance. We expressed *ap^dsRNAi^* in adults until A20 using *ap^GAL4^* (a strong hypomorphic allele) and found that Dimm immunoreactivity was significantly downregulated to 58.5%±6.6 of control (Figure 4I–4K). In contrast, we found that Dimm expression in adult Tv4 neurons was entirely unaffected by *eya^dsRNAi^* (Figure 4F–4H). These data indicate that Dimm becomes independent of *eya* in adult Tv4 neurons, even though *eya* expression persists in adults and *eya* is absolutely required for the initiation of *dimm* expression [9]. We conclude that maintenance of *dimm* remains dependent on *ap* but becomes independent of *eya* and *grh* post-developmentally.

We also examined all other potential cross-regulatory relationships within the Tv4 subtype transcription network (Table S3), but found no instances of a transcription factor requiring the presence of another for its maintenance.

### Maintenance of subtype transcription network output, but not configuration, in mature Tv1 neurons

Is the maintained role found for the Tv4 subtype transcription network common to other subtype transcription networks? To determine this, we examined the output and configuration of the adult Tv1 subtype transcription network. In the neuroblast lineage that gives rise to the Tv1 neuron, *cas* induces *col*. Upon birth of the postmitotic Tv1 neuron *col* initiates *eya* and *ap* expression. Initiation of *dimm* is then absolutely dependent on each of *col*, *ap* and *eya*. Then all four regulators are required for Nplp1 initiation [6], [9].

**Figure 5:**
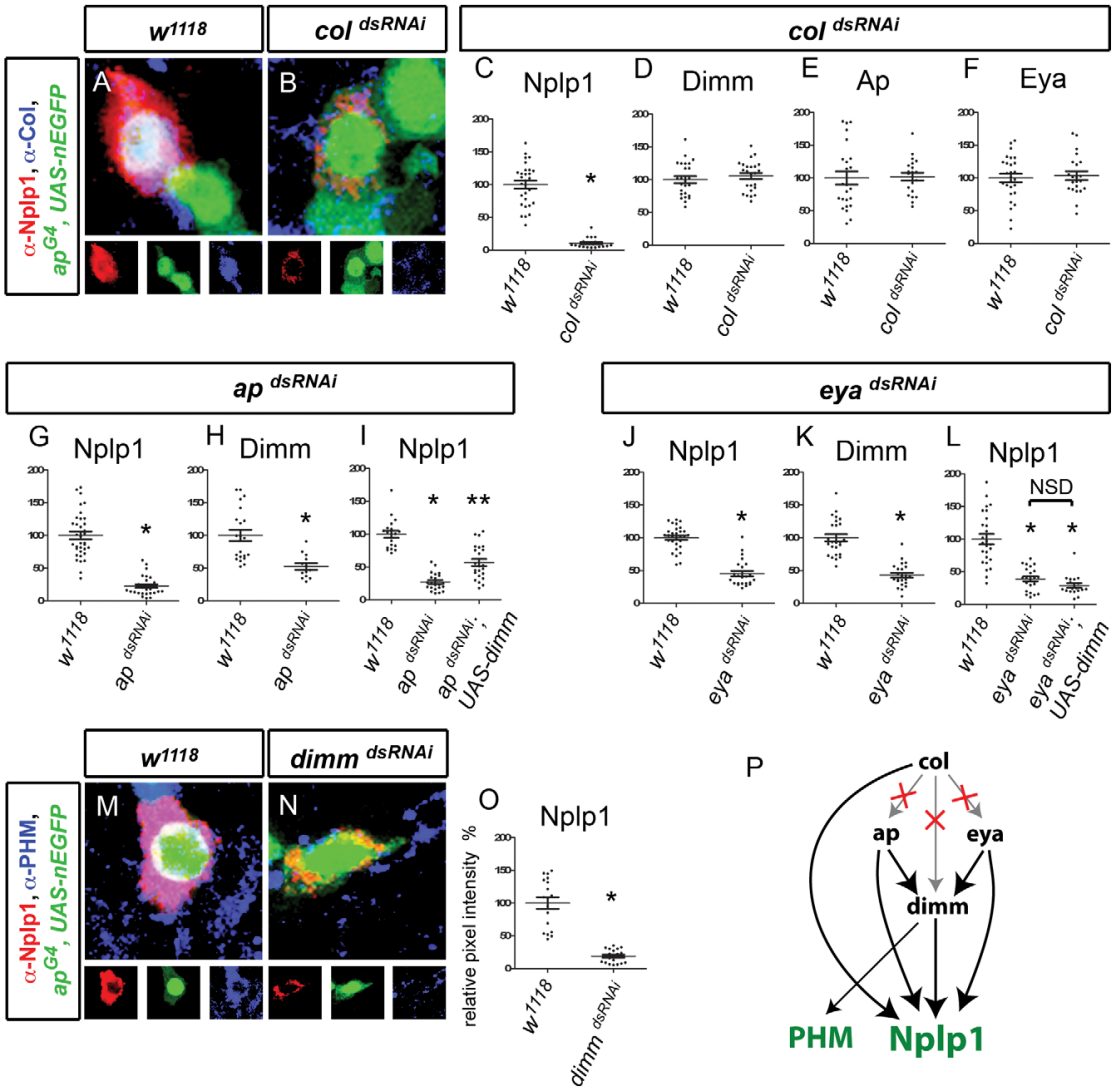
Transcriptional regulation of Nplp1 in adult Tv1 neurons. (A–F) *col^dsRNAi^* downregulated Nplp1 but not *ap*, *eya*, or *dimm* in adult Tv1. (A,B) Expression of Nplp1 (red), *ap^Gal4^,UAS-nEGFP* (green) and Col (blue) in adult Tv4 neurons at A10 at 29°C. Col expression is lost and Nplp1 is downregulated in *col^dsRNAi^* (B) compared to *w^1118^* control (A). (C) Quantification of Nplp1 immunoreactivity in *w^1118^* (n = 29) and *col^dsRNAi^* (n = 20) at A10 at 29°C. (D–F) *col^dsRNAi^* did not affect Dimm immunoreactivity (D), *ap^Gal4^,UAS-nEGFP* fluorescence (E) or Eya immunoreactivity (F) compared to *w^1118^* at A10 at 29°C. *col^dsRNAi^* (D n = 24; E n = 22; F n = 21). *w^1118^* control (D n = 25; E n = 27; F n = 24). (G–I) *ap^dsRNAi^* significantly reduced Nplp1 (G) and Dimm (H) immunoreactivity compared to *w^1118^* at A20 at 29°C. (G) * p<0.0001 *ap^dsRNAi^* (n = 30) compared to *w^1118^* control (n = 36). (H) * p<0.001 *ap^dsRNAi^* (n = 13) compared to *w^1118^* control (n = 22) (I) Dimm restoration *(UAS-dimm)* in *ap^dsRNAi^* background only partially rescued Nplp1 downregulation at A15 at 29°C. * p<0.0001 *ap^dsRNAi^* (n = 22) compared to *w^1118^* control (n = 19). ** p<0.0001 *ap^dsRNAi^;UAS-dimm* (n = 23) compared to *ap^dsRNAi^* and *w^1118^* controls. (J–L) *eya^dsRNAi^* significantly reduced Nplp1 (J) and Dimm (K) immunoreactivity compared to *w^1118^* at A10 at 29°C. (L) Dimm restoration *(UAS-dimm)* in *eya^dsRNAi^* background failed to rescue Nplp1 immunoreactivity at A15 at 29°C. * p<0.0001 *eya^dsRNAi^* and *eya^dsRNAi^*; *UAS-dimm* (n = 19) compared to *w^1118^* control. NSD, no significant difference between *eya^dsRNAi^* and *eya^dsRNAi^*;*UAS-dimm. eya^dsRNAi^* (J n = 23; K n = 23; L n = 22). *w^1118^* control (J n = 30; K n = 25; L n = 26). (M–O) *dimm^dsRNAi^* downregulated Nplp1 and PHM in adult Tv1. (M,N) Tv1 neurons expressing Nplp1 (red), *ap^Gal4^,UAS-nlsEGFP* (green) and PHM (blue) in adult Tv4 neurons at A10 at 29°C. Nplp1 is downregulated and PHM is lost in *dimm^dsRNAi^* (n = 18) (M) compared to *w^1118^* control (n = 15) (N). (O) *dimm^dsRNAi^* significantly reduced Nplp1 immunoreactivity compared to *w^1118^* at A10 at 29°C. * p<0.0001 *dimm^dsRNAi^* (n = 19) compared to *w^1118^* control (n = 18). (P) Model depicting regulation of Nplp1 and PHM and the configuration changes between *ap*, *eya*, *dimm* and *col* from embryonic to adult Tv1. Notably, *col* no longer regulates *dimm*, *eya* or *ap* expression (denoted by X). *Genotypes: w^1118^* (*UAS-dicer2/+; ap^Gal4^/+; tub-Gal80^TS^, UAS-nEGFP/+*); *col^dsRNAi^* (*UAS-dicer2/+; ap^Gal4^/+; tub-Gal80^TS^, UAS-nEGFP/UAS-col^dsRNAi^*); *ap^dsRNAi^* (*UAS-dicer2/+; ap^Gal4^/+; tub-Gal80^TS^, UAS-nEGFP/UAS-ap^dsRNAi^*); *ap^dsRNAi^; UAS-dimm* (*UAS-dicer2/+; ap^Gal4^/UAS-dimm; tub-Gal80^TS^, UAS-nEGFP/UAS-ap^dsRNAi^*); *eya^dsRNAi^* (*UAS-dicer2/UAS-eya^dsRNAi^; ap^Gal4^/+; tub-Gal80^TS^, UAS-nEGFP/+*); *eya^dsRNAi^*; *UAS-dimm* (*UAS-dicer2/UAS-eya^dsRNAi^; ap^Gal4^/UAS-dimm; tub-Gal80^TS^, UAS-nEGFP/+*); *dimm^dsRNAi^* (*UAS-dicer2/+; ap^Gal4^/UAS-eya^dsRNAi^; tub-Gal80^TS^, UAS-nEGFP/+*); *rev4,dimm^dsRNAi^* (*UAS-dicer2/+; ap^Gal4^/rev4, UAS-dimm^dsRNAi^; tub-Gal80^TS^, UAS-nEGFP/+*).

In adult Tv1 neurons, *col*, *eya*, *ap* and *dimm* were maintained (Figure 1). Induction of *col^dsRNAi^* [9] at A1 reduced Nplp1 to 10.7±1.7% of control by A15 (Figure 5A–5C). We also found that induction of *ap^dsRNAi^*, *eya^dsRNAi^* or *dimm^dsRNAi^* significantly reduced Nplp1 expression levels to 22.7±2.7%, 45.3±4.2% and 19.0±2.4% of control, respectively (all p<0.0001 to control) (Figure 5G, 5J, 5O). We verified that Col, Eya and Dimm immunoreactivity in Tv1 were eliminated by their respective *dsRNAi* (Table S4). In addition, we found that *dimm^dsRNAi^* also eliminated PHM expression in Tv1 neurons (Figure 5N). Thus, the Tv1 subtype transcription network is required to maintain the expression of Tv1-specific terminal differentiation gene expression.

Next, we examined the configuration of the adult Tv1 subtype transcription network. Intriguingly, even through col is essential for *eya*, *ap* and *dimm* expression in the embryo, *col^dsRNAi^* had no effect on *dimm*, *ap*, or *eya* expression in Tv1 (Figure 5D–5F). In contrast, expression of either *ap^dsRNAi^* or *eya^dsRNAi^* led to a significant downregulation of Dimm levels in Tv1 to 52.4±5.1% and 42.9±3.7% (Figure 5H, 5K). These data are highly intriguing. The loss of *col*-dependence of *ap*, *eya* and *dimm* on *col* was unexpected, notably because Nplp1 retains its *col*-dependence. Moreover, it is intriguing that *dimm* retains its *eya*-dependence in adult Tv1 neurons, but not in adult Tv4 neurons. Thus, the cross-regulatory interactions between persistent transcription factors can be significantly altered after the process of differentiation.

Genetic studies in the embryo had established that *col*, *ap*, *eya* and *dimm* act non-redundantly to initiate Nplp1 expression during development [6]. We tested whether these transcription factors also act non-redundantly in the adult. As *col^dsRNAi^* dramatically downregulated Nplp1 but did not affect *ap*, *eya* or di*m*m, we conclude that *col* acts non-redundantly in this case. However, *dimm* is dependent on both *ap* and *eya* in Tv1. Therefore, to test for redundancy between these transcription factors, we restored *dimm* (*UAS-dimm*) in either *ap^dsRNAi^* or *eya^dsRNAi^* backgrounds. *UAS-dimm* expression was found to only partially rescue Nplp1 expression in an *ap^dsRNAi^* background, from 22.7±2.7% to 57.0±5.4% (p<0.0001 compared to *ap^dsRNAi^* and also *w^1118^* control) (Figure 5I). However, *UAS-dimm* expression failed to rescue Nplp1 expression in an *eya^dsRNAi^* background (Figure 5L). Thus, as during development, all regulators are required combinatorially for normal Nplp1 expression in adult Tv1 neurons.

### Adult neurons respond predictively to ectopic reconstitution of subtype transcription network activity

**Figure 6:**
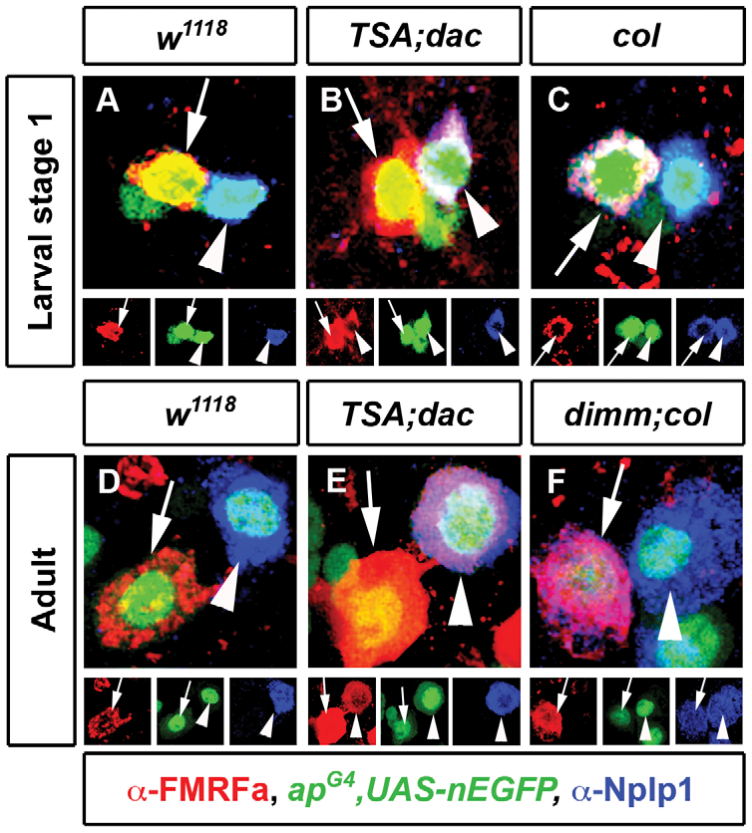
Differentiation networks have different abilities to activate ectopic gene expression in adult neurons. (A–F) Representative images of Tv4 neurons (arrows) and Tv1 neurons (arrowheads) at larval stage 1 (A–C) and adults (D–F). (B,E) Induction of ectopic FMRFa expression (arrowhead, red) in Tv1 (identified with Nplp1, blue) after misexpression of *dac* and constitutively-activated Thickveins and Saxophone BMP receptors (*TSA*) in embryos (B n = 33) and for 5 days in adults (E n = 31). (C) Misexpression of *col* in embryonic Tv neurons using *ap^Gal4^* initiates ectopic Nplp1 expression (arrow, blue) in Tv4 neurons (identified with FMRFa, red) (C n = 24). (F) Misexpression of *col* and upregulation of *dimm* for 5 days in adults using *ap^Gal4^* initiates ectopic Nplp1 expression (arrow, blue) in Tv4 neurons (identified with FMRFa, red) (F n = 42). Genotypes: (A–C) Larval stage 1 *w^1118^* (*ap^Gal4^/+; UAS-nEGFP/+*); *TSA;dac* (*ap^Gal4^/UAS-tkv^A^, UAS-sax^A^; UAS-nEGFP/UAS-dac*); *col* (*ap^Gal4^/UAS-col; UAS-nEGFP/+*). (D-F) Adult *w^1118^* (*ap^Gal4^/+; tub-Gal80^TS^, UAS-nEGFP/+*); *TSA;dac* (*ap^Gal4^/UAS-tkv^A^, UAS-sax^A^; tub-Gal80^TS^, UAS-nEGFP/UAS-dac*); *dimm;col* (*ap^Gal4^/UAS-col; tub-Gal80^TS^, UAS-nEGFP/UAS-dimm*).

During development, late-acting subtype transcription networks can override earlier-acting transcriptional codes to dominantly activate ectopic expression of their target genes and/or subtype identity [6]. For example, in embryos, overexpression of *col* in all Tv neurons ectopically activated Nplp1 in Tv4, presumably reconstituting the *col/ap/eya/dimm* Tv1 subtype transcription network (Baumgardt et al., 2007). Interestingly, this did not disrupt native FMRFa expression in Tv4 neurons, nor its known subtype transcription network profile. Here, we verify these data in embryos (Figure 6A, 6C). Additionally, we demonstrate here that the reciprocal subtype transcription network reconstitution, *dac* and BMP activation in all Tv neurons, is sufficient to initiate FMRFa expression in embryonic Tv1 neurons. Interestingly, we found that this also occurred without a concomitant disruption of Nplp1 expression in Tv1 (Figure 6A, 6B).

We tested whether Tv1 and Tv4 subtype transcription networks retained this capacity in adult neurons. This would prove that subtype transcription networks are capable of inducing expression of their pertinent target gene in a mature cell that never had expressed that gene. We found that activation of BMP signaling and *dac* in adult Tv1 neurons for 5 days robustly activated ectopic FMRFa expression in 100% of Tv1 neurons (Figure 6D, 6E), without affecting Nplp1 in Tv1. We next ectopically expressed *col* in adult Tv neurons, but this failed to induce ectopic Nplp1 expression (n = 21 Tv4 neurons) (data not shown). However, co-expression of *col* and *dimm* in adult Tv neurons was sufficient to trigger ectopic Nplp1 expression in 100% of Tv4 neurons (n = 42) (Figure 6D, 6F). These data show that subtype transcription networks are sufficient to initiate pertinent target gene expression, even in adult neurons that had never expressed the gene.

## Discussion

Our data provide novel insight supporting the view of Blau and Baltimore [26] that cellular differentiation is a persistent process that requires active maintenance, rather than being passively ‘locked-in’ or unalterable. We make two primary findings regarding the long-term maintenance of neuronal identity. First, we find that all known developmental transcription factors acting in postmitotic Tv1 and Tv4 neurons to initiate the expression of subtype terminal differentiation genes are then persistently required to maintain their expression. Second, we found that key developmental cross-regulatory relationships that initiated the expression of certain transcription factors were no longer required for their maintained expression in adults. Notably, we found this to be the case even between transcription factors whose expression persists in adults.

**Figure 7:**
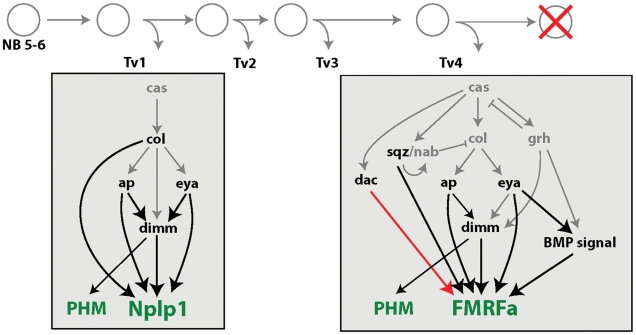
Summary of changes in subtype transcription network configuration between initiation and maintenance of subtype identity. Following terminal differentiation, Tv1 and Tv4 maintain expression of terminal differentiation genes (green text). Tv1 (PHM and Nplp1). Tv4 (PHM and FMRFa). The expression of transcription factors in grey text is lost by larval stages, all transcription factors in black text are retained in adult Tv1/4 neurons. Notably, all black transcription factors have been implicated in differentiation of subtype-specific neuropeptide expression in postmitotic Tv1/4 neurons. In contrast, all grey transcription factors have been implicated in the specification of Tv neuron subtype fates, and not in direct differentiation of subtype neuropeptide gene expression. All remaining transcription factors are required for maintained Nplp1 and FMRFa expression (black arrows), but their cross-regulatory relationships are mostly lost in adults (grey arrows). The only transcription factors that maintain their embryonic configuration are *ap* and *eya's* requirement for *dimm* in Tv1, and *eya's* continued regulation of active BMP signaling in Tv4. Dac plays an enhanced role in FMRFa expression in adult Tv4 neurons (red arrow).

In this study, all transcription factors implicated in the initiation of subtype-specific neuropeptide expression in Tv1 and Tv4 neurons were found to maintain subtype terminal differentiation gene expression in adults (summarized in Figure 7). In Tv1, *col*, *eya*, *ap* and *dimm* are required for Nplp1initiation during development (Figure 1A). In this study, knockdown of each transcription factor in adult Tv1 neurons was shown to dramatically downregulate Nplp1. In Tv4 neurons, FMRFa initiation during development requires *eya*, *ap*, *sqz*, *dac*, *dimm* and retrograde BMP signaling (Figure 1B). Together with our previous work showing that BMP signaling maintains FMRFa expression in adults [14], we now demonstrate that all six regulatory inputs are required for FMRFa maintenance. Most transcription factors, except for *dac*, also retained their relative regulatory input for FMRFa and Nplp1 expression. In addition, individual transcription factors also retained their developmental subroutines. For example, as found during development [10], [11], [20], *dimm* was required in adults to maintain PHM (independently of other regulators) and FMRFa/Nplp1 expression (combinatorially with other regulators).

The few genetic studies that test a persistent role for developmental transcription factors support their role in initiating and maintaining terminal differentiation gene expression. In *C. elegans*, where just one or two transcription factors initiate most neuronal subtype-specific terminal differentiation genes, they then also appear to maintain their target terminal differentiation genes. In ASE and dopaminergic neurons respectively, CHE-1 and AST-1 initiate and maintain expression of pertinent subtype-specific terminal differentiation genes [27], [28]. In vertebrate neurons, where there is increased complexity in the combinatorial activity of transcription factors in subtype-specific gene expression, certain transcription factors have been demonstrated to be required for maintenance of subtype identity. These are Hand2 that initiates and maintains tyrosine hydroxylase and dopa β-hydroxylase expression in mouse sympathetic neurons [29], Pet-1, Gata3 and Lmx1b for serotonergic marker expression in mouse serotonergic neurons [30], [31], and Nurr1 for dopaminergic marker expression in murine dopaminergic neurons [32].

However, while these studies confirm a role for certain developmental transcription factors in subtype maintenance, it had remained unclear whether the elaborate developmental subtype transcription networks, that mediate neuronal differentiation in *Drosophila* and vertebrates, are retained in their entirety for maintenance, or whether they become greatly simplified. Our analysis of all known subtype transcription network factors in Tv1 and Tv4 neurons now indicates that the majority of a developmental subtype transcription network is indeed retained and required for maintenance. Why would an entire network of transcription factors be required to maintain subtype-specific gene expression? The combinatorial nature of subtype-specific gene expression entails cooperative transcription factor binding at clustered cognate DNA sequences and/or synergism in their activation of transcription. In such cases, our data would indicate that this is not dispensed with for maintaining terminal differentiation gene expression in mature neurons.

How the transcription factors of the subtype transcription networks are maintained is less well understood. An elegant model has emerged from studies in *C. elegans*, wherein transcription factors stably auto-maintain their own expression and can then maintain the expression of subtype terminal differentiation genes [33]. The transcription factor CHE-1 is a key transcription factor that initiates and maintains subtype identity in ASE neurons. CHE-1 binds to a cognate DNA sequence motif (the ASE motif) in most terminal differentiation genes expressed in ASE neurons, as well as in its own c*i*s-regulatory region. Notably, a promoter fusion of the *che-1* transcription factor failed to express in *che-1* mutants, indicative of CHE-1 autoregulation [27], [34]. Similar observations were made for AST-1 [28], and for the cooperatively-acting TTX-3 and CEH-10 transcription factors in AIY neurons [35]. Thus, subtype maintenance in *C. elegans* is anchored by auto-maintenance of the transcription factors that initiate and maintain terminal differentiation gene expression.

In contrast, all available evidence in Tv1 and Tv4 neurons fails to support such an autoregulatory mechanism. An *ap* reporter (*apC-τ-lacZ*) is expressed normally in *ap* mutants [12], [36], and in this study *ap^dsRNAi^* was not found to alter *ap^GAL4^* reporter activity (Figure 2I). Moreover, *col* transcription was unaffected in *col* mutants that express a non-functional Col protein [9]. This leaves unresolved the question of how the majority of the transcription factors are stably maintained. For transcription factors that are initiated by transiently expressed inputs, a shift to distinct maintenance mechanisms have been invoked and in certain cases shown [35]. In this study, this was found for the loss of *cas* expression in Tv1 (required for *col* initiation) and the loss of *cas*, *col* and *grh* in Tv4 (required for *eya*, *ap*, *dimm*, *sqz*, *dac* initiation). However, we were surprised to find that the cross-regulatory relationships between persistently-expressed transcription factors were also significantly altered in adults. Notably, *eya* initiated but did not maintain *dimm* in Tv4. In Tv1, *col* initiated but did not maintain *eya*, *ap* or *dimm*. This was particularly unexpected as *eya* remained critical for FMRFa maintenance and *col* remained critical for Nplp1 maintenance. Indeed, although we tested for cross-regulatory interactions between all transcription factors in both the Tv1 and Tv4 subtype transcription networks, only Dimm was found to remain dependent upon its developmental input; Eya and Ap in Tv1 as well as Ap in Tv4. However, even in this case, the regulation of Dimm was changed; it no longer required *eya* in Tv4, and in Tv1 it no longer required *col*, in spite of the fact that both *col* and *eya* are retained in these neurons. We anticipate that such changes in transcription factor cross-regulatory relationships will be found in other *Drosophila* and vertebrate neurons, which exhibit high complexity in their subtype transcription networks [1], [5]. Indeed, recent evidence has found that in murine serotonergic neurons, the initiation of Pet-1 requires Lmx-1b, but ablation of Lmx-1b in adults did not perturb the maintenance of Pet-1 expression [31].

We are pursuing the potential role of autoregulation for the other factors in the Tv1/Tv4 subtype transcription networks. However, we consider there to be three additional, potentially overlapping, models for subtype transcription network maintenance. First, regulators may act increasingly redundantly upon one another. Second, unknown regulators may become increasingly sufficient for transcription factor maintenance. Third, transcription factors may be maintained by dedicated maintenance mechanisms, as has been shown for the role of trithorax group genes in the maintenance of Hox genes and Engrailed [37], [38]. Moreover, chromatin modification is undoubtedly involved and likely required to maintain high-level transcription of Tv transcription factors as well as FMRFa, Nplp1 and PHM. However, the extent to which these are instructive as opposed to permissive has yet to be established [39]. In this light, it is intriguing that MYST-HAT complexes, in addition to the subtype transcription factors Che-1 and Die-1, are required for maintenance of ASE-Left subtype identity in *C. elegans* [31].

Taken together, our studies have identified two apparent types of maintenance mechanism that are operational in adult neurons. On one hand, there are sets of genes that are maintained by their initiating set of transcription factors. These include the terminal differentiation genes and the transcription factor *dimm*. On the other, most transcription factors appear to no longer require regulatory input from their initiating transcription factor(s). Further work will be required to better understand whether these differences represent truly distinct modes of gene maintenance or reflect the existence of yet unidentified regulatory inputs onto these transcription factors. One issue to consider here is that the expression of certain terminal differentiation genes in neurons, but perhaps not subtype transcription factors, can be plastic throughout life, with changes commonly occurring in response to a developmental switch or physiological stimulus [40], [41], [42]. Thus, terminal differentiation genes may retain complex transcriptional control in order to remain responsive to change. It is notable, however, that FMRFa, Nplp1 and PHM appear to be stably expressed at high levels in Tv1/4 neurons, and we have not found any conditions that alter their expression throughout life. Thus, we consider these to be stable terminal differentiation genes akin to serotonergic or dopaminergic markers in their respective neurons that define those cells' functional identity and, where tested, are actively maintained by their developmental inputs [30], [32]. Tv1/4 neurons undoubtedly express a battery of terminal differentiation genes, and sets of unknown transcription factors are likely required for their subtype-specific expression. We consider subtype transcription networks to encompass all regulators required for differentiating the expression of all subtype-specific terminal differentiation genes. Further, we view differentiation of subtype identity as the completion of a multitude of distinct gene regulatory events in which each gene is regulated by a subset of the overall subtype transcription network. As highly restricted terminal differentiation genes expressed in Tv1 and Tv4 neurons, we believe that Nplp1, FMRFa and PHM provide a suitable model for the maintenance of overall identity, with the understanding that other unknown terminal differentiation genes expressed in Tv1 and Tv4 may not be perturbed by knockdown of the transcription factors tested herein. In the future, it will be important to incorporate a more comprehensive list of regulators and terminal differentiation genes for each neuronal subtype. However, we believe that the principles uncovered here for FMRFa, Nplp1 and PHM maintenance will hold for other terminal differentiation genes.

Finally, we propose that the active mechanisms utilized for maintenance of subtype differentiation represent an Achilles heel that renders long-lived neurons susceptible to degenerative disorders. Nurr1 ablation in adult mDA neurons reduced dopaminergic markers and promoted cell death [32]. Notably, Nurr1 mutation is associated with Parkinson's disease [43], and its downregulation is observed in Parkinson's disease mDA neurons [44]. Adult mDA are also susceptible to degeneration in *foxa2* heterozygotes, another regulator of mDA neuron differentiation that is maintained in adult mDA neurons [45]. Studies in other long-lived cell types draw similar conclusions. Adult conditional knockout of Pdx1 reduced insulin and β-cell mass [46], [47] and, importantly, heterozygosity for *Pdx1* leads to a rare monogenic form of non-immune diabetes, MODY4 [48]. Similarly, *NeuroD1* haploinsufficiency is linked to MODY6 [49] and adult ablation of NeuroD in β-islet cells results in β-cell dysfunction and diabetes [50]. These data, together with our results here, underscore the need to further explore the transcriptional networks that actively maintain subtype identity, and hence the function, of adult and aging cells.

## Materials and Methods

### Fly stocks

Flies were maintained on standard cornmeal food and maintained at stable temperatures in environment rooms set at 70% humidity at 18°C, 25°C or 29°C.

### Fly strains

*apterous^md544^* (referred to as *ap^GAL4^*); *ap^P44^; sqz^IE^; sqz^GAL4^; UAS-thickveins activated* (*UAS-tkv^A^*); *UAS-saxophone activated* (*UAS-sax^A^*) [12]; *dac^GAL4^* [51]; *rev4; UAS-dimm* [18]; *dac^3^* [52]; ; *eya^CliIID^* [53]; *grh^GAL4^* [6]. *tubP>GAL80^TS^* (temperature-sensitive GAL80 under the control of the *Drosophila* tubulin 84B promoter) (McGuire et al., 2003); *UAS-nEGFP* (nuclear localized EGFP); *UAS-dicer2* [21].

### *dsRNAi* lines

Strains used for primary data: *UAS-col#24E* [9]; *UAS-ap^dsRNAi^ 8376R-2; UAS-dac^dsRNAi^ 4952R-2*; *UAS-sqz^dsRNAi^ 5557R-2* (NIG-FLY); *UAS-dimm^dsRNAi^ GD44470*; *UAS-eya^dsRNAi^ GD43911* (VDRC). Strains used secondarily to verify data: *UAS-dimm^dsRNAi^ KK103356; UAS-eya^dsRNAi^ 108071KK* (VDRC); *UAS-eya^dsRNAi^ JF03160; UAS-dac^dsRNAi^ JF02322* (TRiP).

### Spatial and temporal regulation of transgene expression using the TARGET system

Flies for TARGET-mediated transgene induction were generated by crossing utility flies (*UAS-dicer2/UAS-dicer2; ap^Gal4^; tubP>GAL80^TS^, UAS-nEGFP/SM6-TM6,Tb*) or (*UAS-dicer/UAS-dicer; dac^Gal4^; tubP>GAL80^TS^, UAS-nEGFP/SM6-TM6,Tb*) to *UAS-dsRNAi* (experimental group) or *w^1118^* flies (control). Experiments were performed on resulting progeny bearing appropriate genotypes (screened by loss of *SM6-TM6*, *Tb* balancer chromosome). All experimental and control flies were raised at 18°C until eclosion (hatching from the pupal case). On adult day 1 (A1), flies were switched to 29°C for the duration of the induction period indicated. Throughout the text, we present *dsRNAi* data for the induction period at which we observe the maximal phenotype of Nplp1 and FMRFa expression. Further details for each *dsRNAi* are provided in the text.

### Antibodies

#### Primary antibodies

Sheep anti-digoxygenin (1∶1500; Roche); rabbit anti-GFP (1∶100; A6455 Invitrogen); rabbit anti-FMRFa (1∶1000; T-4757 Peninsula Labs); mouse anti-β-Galactosidase (1∶100; 40-1a); chicken anti-Nplp1 (1∶1000), guinea pig anti-Dimm (1∶1000) and rat anti-Cas (1∶1000) (gifts from S. Thor, Linkoping U, Sweden); guinea pig anti-Collier (1∶1000; gift from Adrian Moore, RIKEN, Japan); rabbit anti-pMad (1∶100; cell signaling); rabbit anti-nab (gift from Fernando Jimenez Diaz-Benjumea, Universidad Autónoma de Madrid, Spain); mouse anti-Eya (1∶100; clone 10H6) and mouse anti-Dac (1∶2; Mab Dac 2–3) (both from Developmental Studies Hybridoma Bank; Iowa U. Iowa).

#### Secondary antibodies

Donkey anti-sheep Alexa 555 (1∶10; Invitrogen, Carlsbad, USA); donkey anti-mouse Cy5 and donkey anti-rabbit Cy2 (1∶200; Jackson Immunoresearch, West Grove, USA). Antisense DIG-RNA Probe: DIG-Uracil tagged RNA probes were generated using T3 RNA polymerase from clone RH03963 (DGRC: *Drosophila* Genomic Resource Centre, Indiana, USA) containing a 1584 bp FMRFa cDNA (using the Roche DIG-U-RNA Labelling Kit). Probe synthesis was confirmed using gel electrophoresis.

### Multiplex fluorescent in situ hybridization (FISH) and immunohistochemistry

Standard *in situ* and immunohistochemistry protocols were carried out as described [14]. All tissues compared for fluorescence intensity were processed at the same time using the same aliquots of all solutions under the same conditions. They were then mounted on the same slide and confocal settings were calibrated to control staining levels.

### Image and statistical analysis

All images acquired on an Olympus FV1000 confocal microscope. Fluorescent intensity of individual neurons was measured using Image J (US National Institutes of Health). The mean pixel intensity for each neuron was measured from compressed Z-slices, and corrected for background fluorescence. Analysis was performed on every identifiable Tv1 and Tv4 neuron in segments T1 and T3. The resulting value for each Tv neuron was then incorporated as a single datum point towards the mean intensity for each experiment. Each datum point is represented as a percentage of the mean of the *w^1118^* control for that experiment. Data are presented as Mean ± SEM. Representative images of Tv neurons that were directly compared in figures were processed in an identical way, simultaneously, using Adobe Photoshop CS4. Normally distributed unpaired data were compared using a two-tailed T-test assuming equal variance, to identify significant differences between means. All statistical analysis and graphs data were performed using Prism 5 software. (Graphpad).

## Supporting Information

Figure S1 Expression of *grh*, *cas* and *nab* are lost in Tv4 neurons by early L1 stages. (A–C) Representative confocal images of adult Th1 and Th3 Tv clusters (green) in larval stage. Tv4 neurons (arrows) express FMRFa (white), Tv1 neurons (arrowheads) express Nplp1 (blue). Tv1 and Tv4 do not express transcription factors *grh* (A, red), *cas* (B, red) or *nab* (C, red) in larval stages. Flies were maintained at 25°C. Genotype: (A–F) *+/+;FMRFa-LacZ ap^Gal4^; UAS-nEGFP*.
(PDF)

Figure S2 *UAS-ap^dsRNAi^* phenocopies strong *ap* hypomorphs. (A–F) Strong *ap* hypomorphs *ap^Gal4^/ap^RK568^* (B) and *ap^Gal4,^/UAS-ap^dsRNAi^* (C) flies did not develop wings (A–C), and develop the same thoracic dorsal mid line defects (arrows) (D–F). Genotypes: (A,D) *ap^Gal4^/+*; (B,E) *ap^Gal4^/ap^RK568^*; (C,F) *ap^Gal4^/+; UAS-ap-dsRNAi 8376R-2/+*. Flies were incubated at 25°C.
(PDF)

Figure S3 Downregulation of FMRFa by *dac^dsRNAi^* and *dimm^dsRNAi^* lines is enhanced in a heterozygous background for the pertinent transcription factor. Experimental results compare relative pixel intensity of FMRFa peptide (A) and FMRFa transcript (B) of individual Tv4 neurons. Each datum point was normalized to the percentage of the mean of the *w^1118^* control. Data for each genotype is presented as mean ± SEM. * FMRFa levels are significantly different from *w^1118^* control p<0.0001. ** FMRFa levels are significantly different from *dsRNAi* only p<0.001. Genotypes: (A,B) *w^1118^* (*UAS-dicer2/+; ap^Gal4^/+; tub-Gal80ts, UAS-nEGFP/+*). (A) *rev4* (*UAS-dicer2/+; ap^Gal4^/rev4; tub-Gal80ts, UAS-nEGFP/+*); *dimm^dsRNAi^* (*UAS-dicer2/+; ap^Gal4^/UAS-dimm dsRNAi 44470; tub-Gal80ts, UAS-nEGFP/+*); *rev4,dimm^dsRNAi^* (*UAS-dicer2/+; ap^Gal4^/rev4, UAS-dimm dsRNAi 44470; tub-Gal80ts, UAS-nEGFP/+*). (B) *dac4* (*UAS-dicer2/+; ap^Gal4^/dac4; tub-Gal80ts, UAS-nEGFP/+*); *dac^dsRNAi^* (*UAS-dicer2/+; ap^Gal4^/UAS-dac dsRNAi 4952R-2; tub-Gal80ts, UAS-nEGFP/+*); dac4, *dac^dsRNAi^* (*UAS-dicer2/+; ap^Gal4^/dac4, UAS-dac dsRNAi 4952R-2; tub-Gal80ts, UAS-nEGFP/+*).
(PDF)

Table S1 Downregulation of *eya*, *ap* and *sqz* has the same effect on FMRFa peptide as on FMRFa transcript. To verify that downregulation of *eya*, *ap* and *sqz* equally affects FMRFa transcript and peptide levels, we measured the fluorescence intensity of FMRFa peptide following experimental conditions outlined in Figure 2. Table columns: *dsRNAi* line for each TF; Fluorescent intensity for FMRFa in Control and Experimental groups normalized as a percentage of the mean of the control, and presented as mean ± SEM; Sample size where n = number of neurons.
(PDF)

Table S2 Multiple *UAS-dsRNAi* lines targeting different regions of transcription factors downregulate FMRFa immunoreactivity in Tv4. Utility flies (*UAS-dicer2/UAS-dicer2; ap^Gal4^; tub-Gal80^TS^, UAS-nEGFP/SM6-TM6,Tb*) were crossed to various *UAS-dsRNAi* fly lines (experimental group) and *w^1118^* flies (control). F1 generation was raised at 18°C until they eclosed as adults, then kept at 29°C for specified time (Induction time). Table columns: *UAS-dsRNAi* line for each transcription factor; Fluorescence intensity for FMRFa in Control and Experimental groups normalized as a percentage of the mean of the control, and presented as mean ± SEM; Sample size where n = number of neurons; Induction time: duration of time adult flies were maintained at 29°C prior to sampling.
(PDF)

Table S3 Configuration of transcription factor cross-regulation within Tv1 and Tv4 subtype transcription networks. To test the potential effect of the downregulation of each transcription factor on every other transcription factor, we measured transcription factor immunoreactivity or reporter activity following induction of *UAS-dsRNAi* of other transcription factors. Table columns: headings for *UAS-dsRNAi* line for each TF; Fluorescent intensity for each TF in Control and Experimental groups normalized as a percentage of the mean of the control, and presented as mean ± SEM; Sample size where n = number of neurons. Genotypes: *w^1118^* (*UAS –dicer2/+; ap^Gal4^/+; tub-Gal80ts, UAS-nEGFP/+*). *eya^dsRNAi^* (*UAS-dicer2/UAS-eya dsRNAi 43911; ap^Gal4^/+; tub-Gal80ts, UAS-nEGFP/+*). *sqz^dsRNAi^* (*UAS-dicer2/+; ap^Gal4^/+; tub-Gal80ts, UAS-nEGFP/UAS-sqz dsRNAi 5557R-2*). *apP44; ap^dsRNAi^* (*UAS-dicer2/+; ap^Gal4^/apP44; tub-Gal80ts, UAS-nEGFP/UAS-ap dsRNAi 8376R-2*). *dimm^dsRNAi^* (*UAS-dicer2/+; ap^Gal4^/UAS-dimm dsRNAi 44470; tub-Gal80ts, UAS-nEGFP/+*). *dac^dsRNAi^* (*UAS-dicer2/+; ap^Gal4^/UAS-dac dsRNAi 4952R-2; tub-Gal80ts, UAS-nEGFP/+*). *col^dsRNAi^* (*UAS-dicer2/+; ap^Gal4^/+; tub-Gal80ts, UAS-nEGFP/UAS-col dsRNAi#24E*).
(PDF)

Table S4 Configuration of transcription factor cross-regulation within Tv1 and Tv4 terminal selector networks. Utility flies (*UAS-dicer2/UAS-dicer2; ap^Gal4^; tub-Gal80^TS^, UAS-nEGFP/SM6-TM6,Tb*) were crossed to various *UAS-dsRNAi* fly lines (experimental group (expt)) and *w^1118^* flies (control). F1 generation was raised at 18°C until they eclosed as adults, then kept at 29°C for specified time (Induction time). Table columns: *UAS-dsRNAi* line for each transcription factor; Cell counts for the presence of immunoreactivity of the transcription factor being targeted by *dsRNAi* lines in *w^1118^* flies and Experimental groups in Tv1 and Tv4 neurons. Presented as a fraction (cells with immunoreactivity/total number of cells counted). Induction time: duration of time adult flies were maintained at 29°C prior to sampling.
(PDF)
