# Supplementary figures and images for: Developmental Transcriptional Networks Are Required to Maintain Neuronal Subtype Identity in the Mature Nervous System
Source: PLoS Genet. 2012 Feb 23;8(2):e1002501. doi: 10.1371/journal.pgen.1002501 (PMC3285578; doi:10.1371/journal.pgen.1002501)

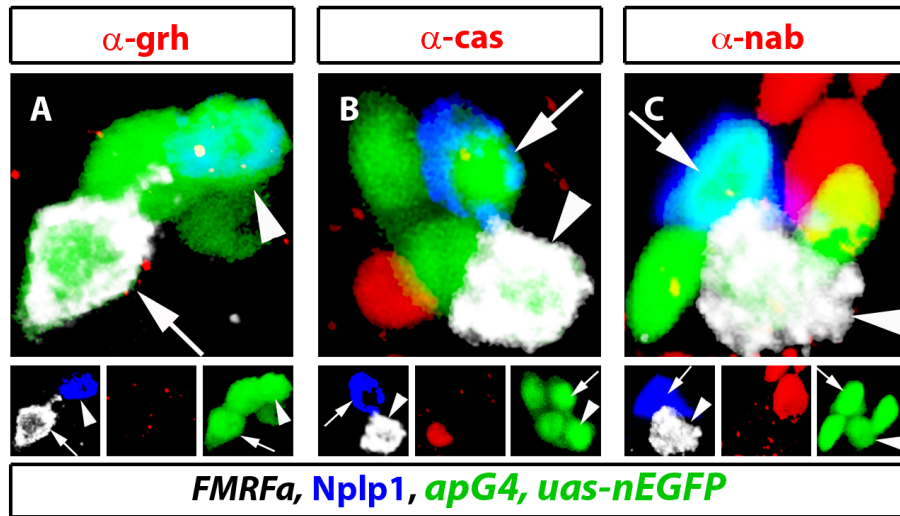

Supplement: Figure S1 — Expression of grh, cas and nab are lost in Tv4 neurons by early L1 stages. (A–C) Representative confocal images of adult Th1 and Th3 Tv clusters (green) in larval stage. Tv4 neurons (arrows) express FMRFa (white), Tv1 neurons (arrowheads) express Nplp1 (blue). Tv1 and Tv4 do not express transcription factors grh (A, red), cas (B, red) or nab (C, red) in larval stages. Flies were maintained at 25°C. Genotype: (A–F) +/+;FMRFa-LacZ apGal4; UAS-nEGFP. (PDF) [file pgen.1002501.s001.pdf]

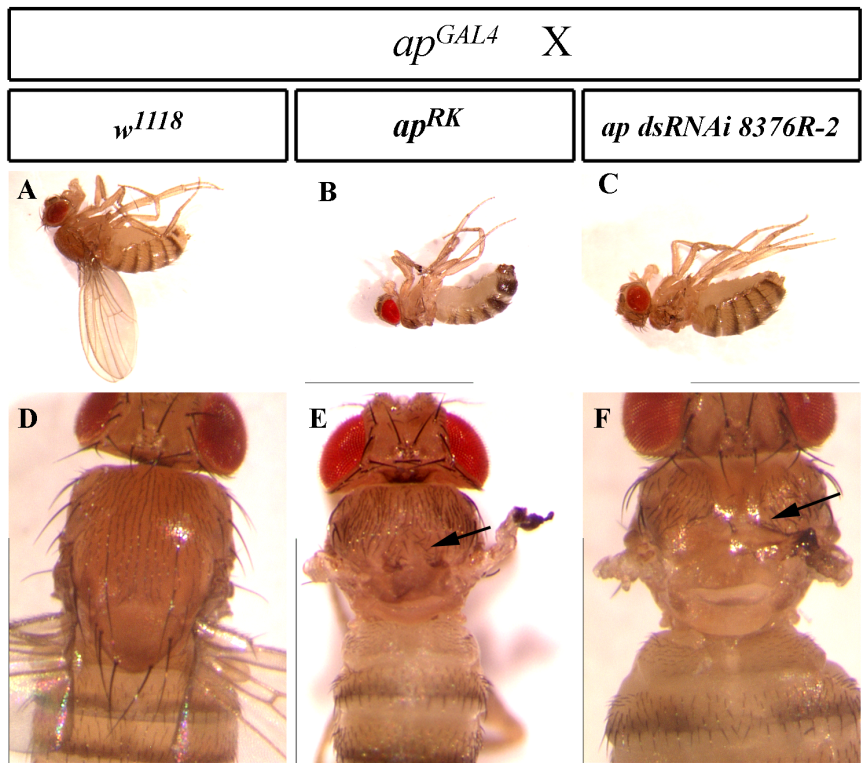

Supplement: Figure S2 — UAS-apdsRNAi phenocopies strong ap hypomorphs. (A–F) Strong ap hypomorphs apGal4/apRK568 (B) and apGal4,/UAS-apdsRNAi (C) flies did not develop wings (A–C), and develop the same thoracic dorsal mid line defects (arrows) (D–F). Genotypes: (A,D) apGal4/+; (B,E) apGal4/apRK568; (C,F) apGal4/+; UAS-ap-dsRNAi 8376R-2/+. Flies were incubated at 25°C. (PDF) [file pgen.1002501.s002.pdf]

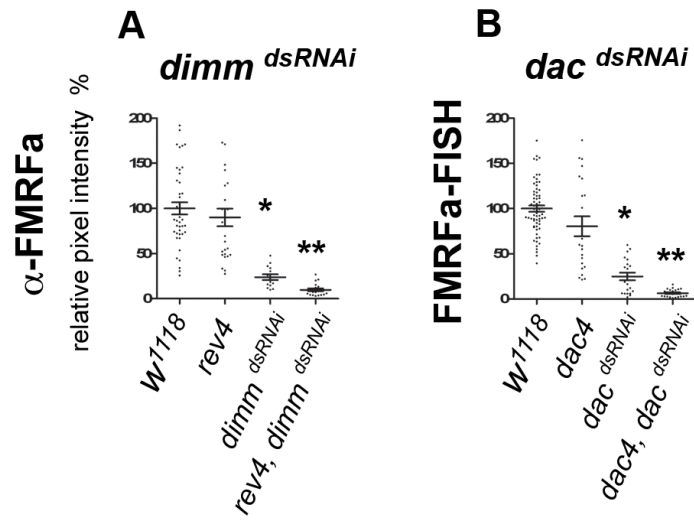

Supplement: Figure S3 — Downregulation of FMRFa by dacdsRNAi and dimmdsRNAi lines is enhanced in a heterozygous background for the pertinent transcription factor. Experimental results compare relative pixel intensity of FMRFa peptide (A) and FMRFa transcript (B) of individual Tv4 neurons. Each datum point was normalized to the percentage of the mean of the w1118 control. Data for each genotype is presented as mean ± SEM. * FMRFa levels are significantly different from w1118 control p<0.0001. ** FMRFa levels are significantly different from dsRNAi only p<0.001. Genotypes: (A,B) w1118 (UAS-dicer2/+; apGal4/+; tub-Gal80ts, UAS-nEGFP/+). (A) rev4 (UAS-dicer2/+; apGal4/rev4; tub-Gal80ts, UAS-nEGFP/+); dimmdsRNAi (UAS-dicer2/+; apGal4/UAS-dimm dsRNAi 44470; tub-Gal80ts, UAS-nEGFP/+); rev4,dimmdsRNAi (UAS-dicer2/+; apGal4/rev4, UAS-dimm dsRNAi 44470; tub-Gal80ts, UAS-nEGFP/+). (B) dac4 (UAS-dicer2/+; apGal4/dac4; tub-Gal80ts, UAS-nEGFP/+); dacdsRNAi (UAS-dicer2/+; apGal4/UAS-dac dsRNAi 4952R-2; tub-Gal80ts, UAS-nEGFP/+); dac4, dacdsRNAi (UAS-dicer2/+; apGal4/dac4, UAS-dac dsRNAi 4952R-2; tub-Gal80ts, UAS-nEGFP/+). (PDF) [file pgen.1002501.s003.pdf]
