# Supplementary material for: Developmental Transcriptional Networks Are Required to Maintain Neuronal Subtype Identity in the Mature Nervous System
Source: PLoS Genet. 2012 Feb 23;8(2):e1002501. doi: 10.1371/journal.pgen.1002501 (PMC3285578; doi:10.1371/journal.pgen.1002501)
Supplement: Table S1 — Downregulation of eya, ap and sqz has the same effect on FMRFa peptide as on FMRFa transcript. To verify that downregulation of eya, ap and sqz equally affects FMRFa transcript and peptide levels, we measured the fluorescence intensity of FMRFa peptide following experimental conditions outlined in Figure 2. Table columns: dsRNAi line for each TF; Fluorescent intensity for FMRFa in Control and Experimental groups normalized as a percentage of the mean of the control, and presented as mean ± SEM; Sample size where n = number of neurons. (PDF) [file pgen.1002501.s004.pdf]

**FMRFa peptide**				
control	n	experimental	n	P value
ap^{P44}; *ap*^{dsRNAi}				
100.0±10.0%	20	65.2±5.4%	16	0.007
eya^{dsRNAi}				
100.0±9.3%	34	22.2±3.6%	38	<0.0001
sqz^{dsRNAi}; *sqz*^{IE}				
100.0±11.4%	15	51.1±11.9%	15	0.008
