# Supplementary material for: Developmental Transcriptional Networks Are Required to Maintain Neuronal Subtype Identity in the Mature Nervous System
Source: PLoS Genet. 2012 Feb 23;8(2):e1002501. doi: 10.1371/journal.pgen.1002501 (PMC3285578; doi:10.1371/journal.pgen.1002501)
Supplement: Table S2 — Multiple UAS-dsRNAi lines targeting different regions of transcription factors downregulate FMRFa immunoreactivity in Tv4. Utility flies (UAS-dicer2/UAS-dicer2; apGal4; tub-Gal80TS, UAS-nEGFP/SM6-TM6,Tb) were crossed to various UAS-dsRNAi fly lines (experimental group) and w1118 flies (control). F1 generation was raised at 18°C until they eclosed as adults, then kept at 29°C for specified time (Induction time). Table columns: UAS-dsRNAi line for each transcription factor; Fluorescence intensity for FMRFa in Control and Experimental groups normalized as a percentage of the mean of the control, and presented as mean ± SEM; Sample size where n = number of neurons; Induction time: duration of time adult flies were maintained at 29°C prior to sampling. (PDF) [file pgen.1002501.s005.pdf]

dsRNAi line	Wild type	n	experimental	n	P value	Induction time
ap^{dsRNAi}						
JF02311	100.0±8.2%	34	61.25±6.7%	29	<0.0001	20days
eya^{dsRNAi}						
JF03160	100.0±8.0%	32	50.6±5.9%	27	<0.0001	20 days
108071KK	100.0±8.0%	32	27.0±4.7%	27	<0.0001	20 days
dac^{dsRNAi}						
JF02322	100.0±6.0%	19	52.9±8.5%	13	<0.0001	15 days
dimm^{dsRNAi}						
103356KK	100.0±5.6%	27	40.8±3.1%	36	<0.0001	15 days
