# Supplementary material for: Developmental Transcriptional Networks Are Required to Maintain Neuronal Subtype Identity in the Mature Nervous System
Source: PLoS Genet. 2012 Feb 23;8(2):e1002501. doi: 10.1371/journal.pgen.1002501 (PMC3285578; doi:10.1371/journal.pgen.1002501)
Supplement: Table S3 — Configuration of transcription factor cross-regulation within Tv1 and Tv4 subtype transcription networks. To test the potential effect of the downregulation of each transcription factor on every other transcription factor, we measured transcription factor immunoreactivity or reporter activity following induction of UAS-dsRNAi of other transcription factors. Table columns: headings for UAS-dsRNAi line for each TF; Fluorescent intensity for each TF in Control and Experimental groups normalized as a percentage of the mean of the control, and presented as mean ± SEM; Sample size where n = number of neurons. Genotypes: w1118 (UAS –dicer2/+; apGal4/+; tub-Gal80ts, UAS-nEGFP/+). eyadsRNAi (UAS-dicer2/UAS-eya dsRNAi 43911; apGal4/+; tub-Gal80ts, UAS-nEGFP/+). sqzdsRNAi (UAS-dicer2/+; apGal4/+; tub-Gal80ts, UAS-nEGFP/UAS-sqz dsRNAi 5557R-2). apP44; apdsRNAi (UAS-dicer2/+; apGal4/apP44; tub-Gal80ts, UAS-nEGFP/UAS-ap dsRNAi 8376R-2). dimmdsRNAi (UAS-dicer2/+; apGal4/UAS-dimm dsRNAi 44470; tub-Gal80ts, UAS-nEGFP/+). dacdsRNAi (UAS-dicer2/+; apGal4/UAS-dac dsRNAi 4952R-2; tub-Gal80ts, UAS-nEGFP/+). coldsRNAi (UAS-dicer2/+; apGal4/+; tub-Gal80ts, UAS-nEGFP/UAS-col dsRNAi#24E). (PDF) [file pgen.1002501.s006.pdf]

Tv4

TF	control	n	experimental	n	P value
			ap^{dsRNAi}		
Eyes absent	100.0±6.4%	11	119.4±9.3%	14	0.12
Dachshund	100.0±8.0%	22	117.0±10.8%	15	0.20
			eya^{dsRNAi}		
Apterous	100.0±8.5%	16	95.1±8.0%	16	0.68
Dachshund	100.0±7.3%	20	95.3±5.7%	22	0.61
			dac^{dsRNAi}		
Apterous	100.0±6.2%	47	109.0±14.6%	24	0.51
Eyes absent	100.0±6.1%	44	82.1±8.5%	26	0.08
Dimmed	100.0±5.6%	35	94.9±8.0%	19	0.60
			dimm^{dsRNAi}		
Apterous	100.0±10.1%	18	102±8.1%	18	0.88
Dachshund	100.0±7.8%	14	95.0±8.0%	11	0.66
Eyes absent	100.0±6.4%	18	166.5±13.4%	18	<0.0001
			sqz^{dsRNAi}; *sqz*^{IE}		
Apterous	100.0±11.4%	13	111.0±9.1%	14	0.46
Dachshund	100.0±7.7%	14	91.2±2.6%	18	0.25
Eyes absent	100.0±15.8%	16	95.2±7.3%	18	0.77
Dimmed	100.0±7.5%	13	114.2±7.5%	14	0.25

Tv1

TF	control	n	experimental	n	P value
			ap^{dsRNAi}		
Eyes absent	100.0±6.5%	16	88.8±7.0%	15	0.24
Collier	100.0±7.2%	17	142.3±9.5%	20	0.25
			eya^{dsRNAi}		
Apterous	100.0±11.3%	17	148.1±16.0%	19	0.02
Collier	100.0±6.4%	17	123.9±8.6%	19	0.03
			dimm^{dsRNAi}		
Apterous	100.0±11.4%	29	92.3±10.3%	23	0.62
Collier	100.0±10.1%	15	107.2±5.9%	15	0.54
Eyes absent	100.0±4.8%	29	122.4±6.3%	23	0.006
