# Supplementary material for: Developmental Transcriptional Networks Are Required to Maintain Neuronal Subtype Identity in the Mature Nervous System
Source: PLoS Genet. 2012 Feb 23;8(2):e1002501. doi: 10.1371/journal.pgen.1002501 (PMC3285578; doi:10.1371/journal.pgen.1002501)
Supplement: Table S4 — Configuration of transcription factor cross-regulation within Tv1 and Tv4 terminal selector networks. Utility flies (UAS-dicer2/UAS-dicer2; apGal4; tub-Gal80TS, UAS-nEGFP/SM6-TM6,Tb) were crossed to various UAS-dsRNAi fly lines (experimental group (expt)) and w1118 flies (control). F1 generation was raised at 18°C until they eclosed as adults, then kept at 29°C for specified time (Induction time). Table columns: UAS-dsRNAi line for each transcription factor; Cell counts for the presence of immunoreactivity of the transcription factor being targeted by dsRNAi lines in w1118 flies and Experimental groups in Tv1 and Tv4 neurons. Presented as a fraction (cells with immunoreactivity/total number of cells counted). Induction time: duration of time adult flies were maintained at 29°C prior to sampling. (PDF) [file pgen.1002501.s007.pdf]

dsRNAi lines	Tv4		
	w^{1118}	expt	days ind
	eya^{dsRNAi}		
43911	36/36	0/26	15d
eyaCliIID; 43911	36/36	0/38	15d
JF03160	40/40	0/27	20 d
108071KK	40/40	025	20 d
	dac^{dsRNAi}		
4952R-2	52/52	0/27	10d
dac3, 4952R-2	52/52	0/41	10d
JF02322	35/35	0/29	15 d
	dimm^{dsRNAi}		
44470	44/44	0/14	10d
rev4,44470	44/44	0/20	10d
103356KK	27/27	0/39	15 d

dsRNAi lines	Tv1		
	w^{1118}	expt	days ind
	col^{dsRNAi}		
#24E	29/29	0/20	10d
	eya^{dsRNAi}		
43911	25/25	0/20	20d
	dimm^{dsRNAi}		
44470	18/18	0/19	10d
rev4,44470	11/11	0/20	10d
